# Loss of Myh11 K1256 Dysregulates the Extracellular Matrix and Focal Adhesion by Inhibiting Zyxin-Activated Transcription

**DOI:** 10.3390/ijms26167853

**Published:** 2025-08-14

**Authors:** Shota Tomida, Hironori Okuhata, Tamaki Ishima, Ryozo Nagai, Kenichi Aizawa

**Affiliations:** 1 Department of Translational Research, Clinical Research Center, Jichi Medical University Hospital, Tochigi 329-0498, Japan; 2 School of Medicine, Faculty of Medicine, Gunma University, Gunma 371-8511, Japan; 3 Jichi Medical University, Tochigi 329-0498, Japan; 4 Clinical Pharmacology Center, Jichi Medical University Hospital, Tochigi 329-0498, Japan

**Keywords:** aortic dissection, proteomics, Myh11

## Abstract

Pathogenic variants of MYH11, which encode smooth muscle myosin heavy chain 11, have been linked to familial thoracic aortic aneurysms and dissections (FTAAD). However, molecular pathways affected by these mutations have not been well understood. To explore downstream consequences of Myh11 disruption, we analyzed transcriptomic and proteomic profiles of aortas from male Myh11 mice with homozygous deletion of lysine 1256 (K1256) and of wild-type controls. Of 6499 proteins quantified, 1763 were differentially expressed (adjusted *p* < 0.05), including 942 that were downregulated and 821 that were upregulated in mutant aortas. Enrichment analysis of downregulated genes and proteins revealed a consistent reduction in extracellular matrix-related pathways. Among downregulated proteins, we identified tenascin Xb, transforming growth factor β (Tgfb) 2, and Tgfb receptor 1/2, malfunctions of which are linked to connective tissue diseases, such as Ehlers–Danlos and Loeys–Dietz syndromes. Nevertheless, unlike these syndromic diseases, mice with Myh11 pathogenic variants and patients with FTAAD do not exhibit syndromic features, likely reflecting expression of Myh11 restricted to smooth muscle. These results suggest that loss of Myh11 disrupts maintenance of extracellular matrix by SMCs, the loss of which contributes to aortic fragility without affecting other tissues.

## 1. Introduction

Aortic dissection is a life-threatening condition arising from an intimal tear in the aortic wall that allows blood to separate layers of the aorta [1]. At least 20% of non-syndromic thoracic aortic disease cases cluster in families, a disorder known as familial thoracic aortic aneurysms and dissections (FTAAD) [2,3,4,5]. Among key genes implicated in FTAAD are those encoding components of the vascular smooth muscle cell (VSMC) contractile apparatus, including *ACTA2*, *MYH11*, *MYLK*, and *PRKG1* [6]. *MYH11*, in particular, encodes the smooth muscle-specific myosin heavy chain, and pathogenic variants of *MYH11* are a known cause of FTAAD [2,7,8]. Pathogenic mutations typically occur in the C-terminal coiled-coil domain, disrupting polymerization of thick filaments and impairing VSMC contractile function [2,6,7]. Notably, we have shown that mice carrying a single lysine deletion in Myh11 (K1256del), a pathogenic variant identified in FTAAD families [8], mimic key disease features [2]. Mice with homozygous K1256del (Myh11^ΔK/ΔK^) exhibited aortic wall thickening and ultrastructural abnormalities, including weakened cell–extracellular matrix (ECM) adhesions [2]. Upon angiotensin II stimulation, mice carrying Myh11 K1256del develop aortic dissection [2]. Mechanistic analyses implicated SMC contractile dysfunction secondary to the Myh11 mutation [2]. Specifically, mutant aortas showed downregulation of integrin subunit α2 (Itga2), a key cell-ECM adhesion receptor, as well as diminished contractile responses to vasomotor stimulation [2]. Complementary multi-omic data further characterized a contractile deficit [9]. Transcriptomic profiling of Myh11^ΔK/ΔK^ aortas revealed reduced expression of calcium transporters, and metabolomic analysis showed blunted activation of the calcium intake pathway due to decreased poly-ADP-ribose production [9]. Collectively, our previous studies have shown that Myh11 K1256del weakens SMC contractility via cell-intrinsic calcium handling and cell-ECM adhesion, thereby predisposing the aorta to mechanical failure under stress.

In addition to VSMC dysfunction, aberrant remodeling of the ECM in the aortic wall contributes to aneurysm and dissection. Aortic medial degeneration, classically defined by fragmentation of elastic fibers, loss of SMCs, and proteoglycan accumulation, is a histopathological hallmark of aortic dissection [10,11]. Recent histopathological analyses confirmed that moderate-to-severe medial degeneration strongly correlates with susceptibility to dissection, whereas mild degeneration is more strongly associated with stable aneurysm formation [12]. The aortic media is a highly organized layer composed of SMCs and elastic lamellae, and its structural integrity depends on balanced remodeling of extracellular matrix components such as elastin, collagens, and proteoglycans, as well as proper SMC function [13]. Aneurysm progression disrupts the structural integrity of the aortic media and adventitia via ECM breakdown [14]. To compensate for ECM breakdown, SMCs assume a synthetic phenotype and produce ECM in a disorganized manner [14]. For example, after elastin degradation, collagen production is increased, which stiffens and thickens the vascular wall [15,16,17].

While significant progress has been made in identifying genetic mutations and transcriptional changes in aortic aneurysm models, we still understand few of the proteomic alterations that ultimately execute tissue-level pathology. In particular, ECM proteins often have long half-lives and may accumulate damage or modifications; therefore, ECM disruption may not be evident at the gene expression level. Unbiased proteomics enable detection of quantitative changes in aortic wall protein composition and identification of protein networks perturbed by pathogenic variants. Thus, in this study, we performed a comprehensive proteomic analysis of aortas from Myh11^ΔK/ΔK^ mice.

## 2. Results

### 2.1. Proteomics

**Figure 1:**
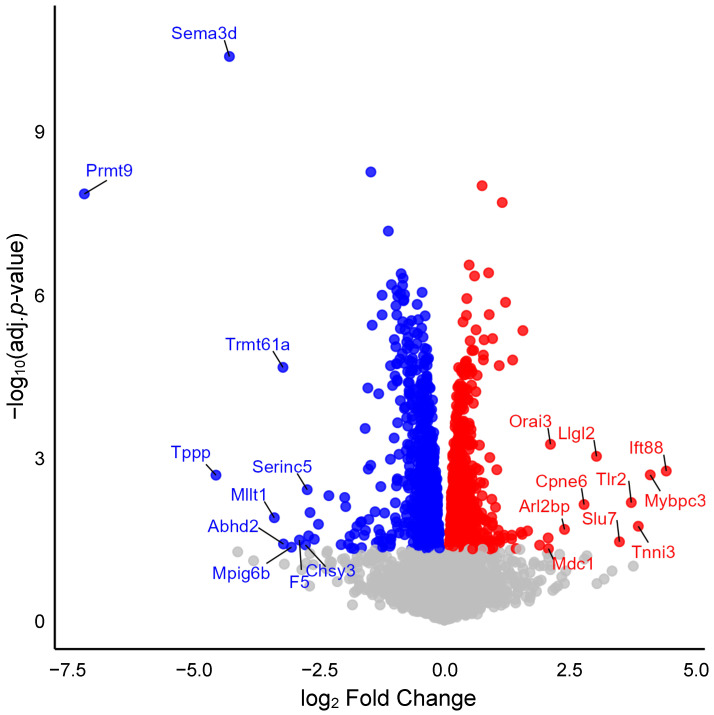
Volcano plot showing differential protein expression. The *x*-axis represents the log_2_ fold change in expression between Myh11^ΔK/ΔK^ (homozygous K1256 deletions of Myh11) and wild-type aortas. The *y*-axis shows log10-adjusted *p*-values. Red dots indicate significantly upregulated proteins, and blue dots indicate significantly downregulated proteins (adjusted *p*-values < 0.05). Gray dots indicate non-significant proteins. Sema3d—Semaphorin-3D, Prmt9—Protein arginine N-methyltransferase 9, Trmt61a—tRNA (adenine(58)-N(1))-methyltransferase catalytic subunit TRMT61A, Tppp—Tubulin polymerization-promoting protein, Serinc5—Serine incorporator 5, Mllt1—Btk-PH-domain binding protein, Abhd2—Monoacylglycerol lipase ABHD2, Mpig6b—Megakaryocyte and platelet inhibitory receptor G6b, F5—Coagulation factor V, Chsy3—Chondroitin sulfate synthase 3, Orai3—Protein orai-3, Llgl2—LLGL scribble cell polarity complex component 2, Ift88—Intraflagellar transport protein 88 homolog, Cpne6—Copine-6, Tlr2—Toll-like receptor 2, Mybpc3—Myosin-binding protein C, cardiac-type, Arl2bp—ADP-ribosylation factor-like protein 2-binding protein, Slu7—Pre-mRNA-splicing factor SLU7, Tnni3—Troponin I, cardiac muscle, Mdc1—Mediator of DNA damage checkpoint protein 1.

We analyzed protein expression in aortic lysates from wild-type and Myh11^ΔK/ΔK^ mice. Of 6499 proteins compared in the two groups, 1730 showed statistically significant differences in expression (adjusted *p*-value < 0.05). Compared to wild-type aortas, Myh11^ΔK/ΔK^ aortas had 922 downregulated and 808 upregulated proteins (Figure 1 and Supplementary Files).

### 2.2. Enrichment of ECM and Focal Adhesion Pathways

**Figure 2:**
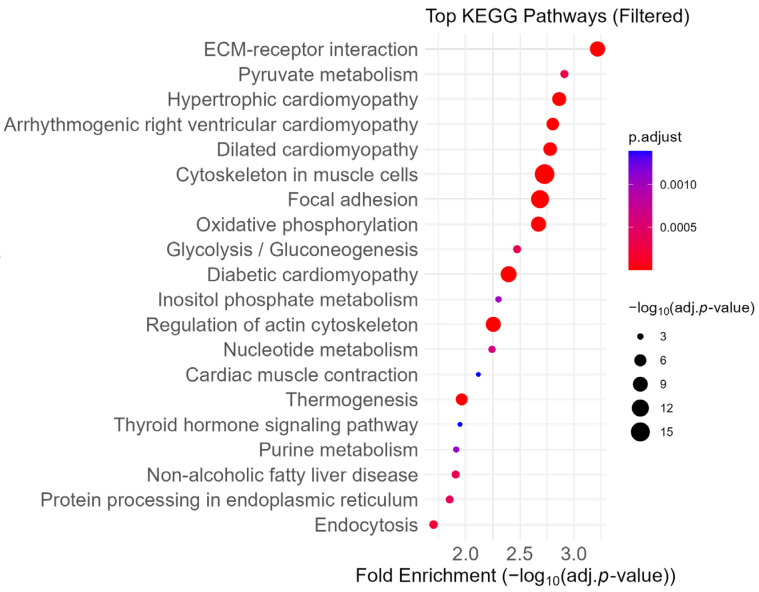
Dot plot of enriched KEGG pathways ranked by fold enrichment. Pathways related to the immune system, neurodegenerative disease, and cancer were excluded to highlight biological processes more specific to smooth muscle cells. Dot size reflects −log_10_ (adjusted *p*-value), and color represents adjusted *p*-value. KEGG—Kyoto Encyclopedia of Genes and Genomes, ECM—Extracellular Matrix.

First, we ran KEGG pathway analysis on our proteomic data. Similar to FSEA, proteins dysregulated in Myh11^ΔK/ΔK^ aortas were highly enriched in extracellular matrix (Figure 2). In agreement with our previous study, proteins related to focal adhesion were also enriched in Myh11^ΔK/ΔK^ aortas. Integrin, a component of focal adhesion, was downregulated (Itga2b, Itgb5, Itga5, Itga3, Itgav, Itga8, Itgb3, and Itgb1: log_2_ fold change = −1.314, −0.7, −0.558, −0.394, −0.38, −0.348, −0.292, and −0.252, respectively; Supplementary Tables S1–S20). These results suggest that the extracellular matrix and focal adhesion were dysregulated.

### 2.3. Attenuation of ECM- and Coagulation-Related Pathways

Since KEGG pathway analysis only accounts for protein enrichment, we next ran fold-change-specific enrichment analysis (FSEA) on our proteomic data using Fold GO to examine relative protein expression levels [18]. Cellular component-related pathways with the highest fold change included the extracellular region and its subcomponents, such as *extracellular matrix*, *collagen-containing extracellular matrix*, and *extracellular space*, followed by *basement membrane*, *cell projection*, and *plasma membrane-bound cell projection* (Table 1 and Supplementary Tables S21–S30). Pathways related to the extracellular matrix, such as the *extracellular matrix structural constituent conferring tensile strength* and the *extracellular matrix structural constituent,* were highly enriched in molecular function sub-ontology as well (Table 2 and Supplementary Tables S31–S40). This suggests that while some proteins related to the extracellular matrix were enriched, others were strongly downregulated in Myh11^ΔK/ΔK^ aortas. By incorporating fold change in pathway analysis, we found that in the biological process sub-ontology, the 13 most altered pathways were related to coagulation, suggesting that Myh11^ΔK/ΔK^ aortas are deficient in coagulation (Table 3 and Supplementary Tables S41–S52). Platelet factors 2, 4, 5, 10, and 13b and thrombomodulin are downregulated (log_2_ fold change = −0.65, −2.89, −0.64, −1.27, and −0.64, respectively); thus, coagulation is probably inactivated.

### 2.4. Enrichment of Upregulated Proteins in Aerobic Respiration- and Ribosome-Related Pathways Revealed by Fold-Change-Specific Enrichment Analysis

Proteins related to aerobic respiration and ribosomes were upregulated. Pathways such as *organellar ribosome*, *organellar large ribosomal subunit*, *mitochondrial ribosome*, and *mitochondrial large ribosomal subunit* were upregulated, suggesting that translation was increased in Myh11^ΔK/ΔK^ mice (Table 4). Enrichment of pathways such as *mitochondrial respiratory chain*, *mitochondrial matrix*, *mitochondrial membrane part*, *respiratory chain*, *inner mitochondrial membrane protein complex*, and *respiratory chain complex* suggests that aerobic respiration was enhanced in Myh11^ΔK/ΔK^ aortas (Table 4 and Supplementary Tables S53–S64). Proteins comprise mitochondrial complex I (Ndufb4, Ndufb5, Ndufv3, Ndufv2, Ndufb8, Ndufb7, Ndufa7, Ndufs7, Ndufa10, and Ndufa12; log_2_ fold change = 0.80, 0.80, 0.78, 0.69, 0.58, 0.57, 0.46, 0.45, and 0.42, respectively) [19], complex II (Uqcrb; log_2_ fold change = 0.89) [20], and complex III (Cox4i1 and Cox5b, Cytb, Cyc1; log_2_ fold change = 0.64, 0.57, 0.46, and 0.41) [20,21], which were upregulated (Supplementary Table S63).

### 2.5. Enriched Pathways Common to Proteomic and Transcriptomic Data

In our previous study, we ran FSEA on transcriptomes of Myh11^ΔK/ΔK^ aortas [9]. We cross-referenced enriched pathways identified by proteomic and transcriptomic analysis and identified 19 attenuated pathways that were common to both proteomic and transcriptomic data (Table 5). Among those enriched pathways, *inorganic molecular entity transmembrane transporter activity*, *cell projection*, and *plasma membrane-bound cell projection* were strongly downregulated in both transcriptomes and proteomes from Myh11^ΔK/ΔK^ aortas (Table 5). Notably, *cell projections* and *plasma membrane-bound cell projections* include proteins forming focal adhesions such as integrins, paxillin, and Rous sarcoma oncogene (Supplementary Tables S27 and S28), suggesting that focal adhesion is attenuated at both protein and transcriptomic levels. No upregulated pathways were common to both proteomic and transcriptomic datasets.

### 2.6. Interaction of Myh11 with Components of Focal Adhesion

**Figure 3:**
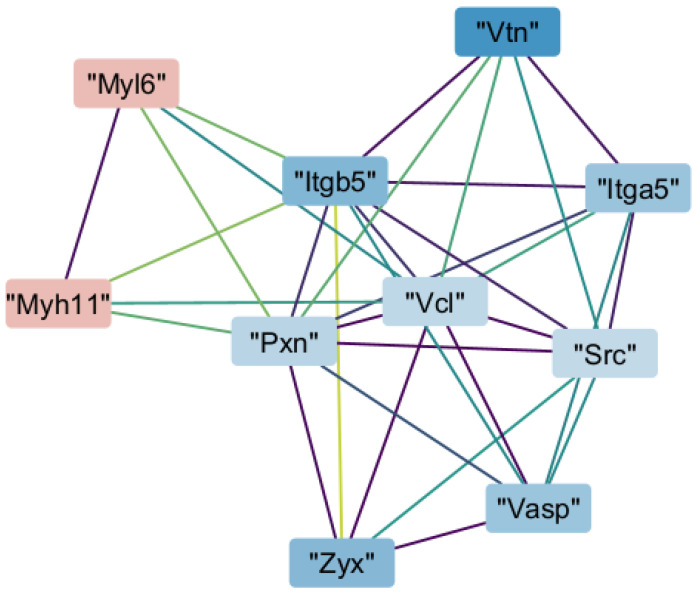
Protein–protein interaction network of a highly connected Molecular Complex Detection (MCODE) cluster. Nodes represent proteins colored by log_2_ fold change (red: upregulated; blue: downregulated). Edges reflect Search Tool for the Retrieval of Interacting Genes/Proteins (STRING)-based interactions, with colors indicating confidence (purple: low; green/yellow: high). Myh11—Myosin heavy chain 11, Myl6—myosin light chain 6; Itgb5—integrin subunit α 5; Itga5—integrin subunit β 5; Pxn—paxillin; Vcl—vinculin; Src—Rous sarcoma oncogene; Vasp—vasodilator-stimulated phosphoprotein; Vtn—vitronectin; Zyx—zyxin.

We then analyzed our proteomic dataset based on interactions of each protein with others. We identified four clusters of protein–protein interactions that scored more than 5 out of 10 in the MCODE application of Cytoscape version 3.10.3. The cluster with the highest score (7.111) indicated that Myh11 interacted with proteins involved in focal adhesion. Myh11 directly interacted with paxillin, integrin subunit β5, and vinculin. Through those proteins, Myh11 indirectly interacted with vinculin, integrin subunit α5 (Itga5), Rous sarcoma oncogene, vasodilator-stimulated phosphoprotein, and zyxin (Figure 3).

### 2.7. Transcription Promoted by Zyxin

Zyxin promotes gene expression in vascular cells by nuclear translocation of itself or yes-associated protein (YAP) after experiencing stretching stimuli [22]. Thus, we sought to identify proteins downregulated by decreased zyxin expression. Using a publicly available microarray dataset [23], we found 621 genes whose expression was lower in zyxin knockout VSMCs than in wild-type VSMCs after being stretched. Of those, 51 genes were also downregulated in Myh11^ΔK/ΔK^ aortas (Supplementary Table S65). Since those genes/proteins were downregulated in stretched zyxin knockout VSMCs, transcription of those genes was likely to be directly regulated by zyxin. We investigated whether those genes belong to KEGG pathway and GO terms that were enriched in Myh11^ΔK/ΔK^ aortas. We found that Itga5 and syndecan 4 belong to the *ECM-receptor interaction* (KEGG ID: mmu04512) and *cytoskeleton in muscle cells* (mmu04820). Itga5 and platelet-derived growth factor receptor β belong to *focal adhesion* (mmu04510). None of the 51 genes belong to *Oxidative phosphorylation* (mmu00190) or *Pyruvate metabolism* (mmu00620). RAS p21 protein activator 3 and solute carrier family 9 member A6 belong to the *inorganic molecular entity transmembrane transporter activity* (GO:0015318). Neuroplastin and solute carrier family 9 member A6 belong to *cell projection* (GO:0042995) and *plasma membrane-bound cell projection* (GO:0120025). Syndecan 4 belongs to the *response to wounding* (GO:0009611). In summary, zyxin regulates at least three KEGG pathways and three GO terms including those related to ECM and focal adhesion.

### 2.8. Downregulation of Proteins Whose Malfuntion Causes Loeys–Dietz and Ehlers–Danlos Syndromes

Since pathway analyses implicated ECM dysregulation in Myh11^ΔK/ΔK^ aortas, we further examined proteins associated with connective tissue diseases. We evaluated expression of 33 proteins that cause connective tissue diseases [24]. Tenascin Xb (Txb), a protein whose haploinsufficiency or pathogenic variants are associated with Ehlers–Danlos syndrome, was downregulated [24,25]. In addition, transforming growth factor β2 (Tgfβ2) and Tgfβ receptors 1 and 2, which are mutated in Loeys–Dietz syndrome, were also downregulated, with log_2_ fold changes of –0.31, –0.41, and –0.13, respectively [26,27]. These findings suggest that Myh11^ΔK/ΔK^ aortas may share pathogenic mechanisms with Ehlers–Danlos syndrome and Loeys–Dietz syndrome, particularly in regard to aortic dissection susceptibility.

## 3. Discussion

In the present study, we employed proteomic analysis to characterize alterations in protein expression and pathway enrichment in aortas from Myh11^ΔK/ΔK^ mice. Our findings suggest decreased signaling related to extracellular matrix composition and focal adhesion in Myh11^ΔK/ΔK^ aortas and increased mitochondrial activity and translation. These findings mirror transcriptomic trends from our previous research and add deeper insights into the pathophysiology of FTAAD by integrating previous studies.

Our previous study indicated focal adhesion disruption in Myh11^ΔK/ΔK^ aortas due to *Itga2* downregulation [2]. Study of R247C, another pathogenic variant of Myh11, also showed disruption of focal adhesion [28]. Pathway analysis during this study expanded that observation, finding that focal adhesion signaling was decreased at the protein level. Furthermore, protein–protein interaction network analysis revealed that paxillin, vinculin, and Itgb5, component proteins of focal adhesion, interact directly with Myh11. In addition, five other proteins that participate in focal adhesion, including zyxin, were shown to interact with Myh11.

Despite previous advances in the understanding of Myh11 pathophysiology, we still do not know how cytoskeletal protein dysfunction leads to altered expression of numerous proteins. Based on the findings of this study, one possible explanation is that transcriptional activation by zyxin is attenuated in Myh11^ΔK/ΔK^ aortas. A previous study demonstrated that zyxin is phosphorylated in response to activation of transient receptor potential cation channel subfamily C member 3 (TRPC3) [29]. Stretch stimulation of SMCs triggers the release of endothelin (ET) 1 mediated by TRPC3 [29]. ET1 promotes atrial natriuretic peptide (ANP) release [29]. ANP activates guanylate cyclase A, which converts GTP to cyclic GMP [29]. Binding of cGMP activates protein kinase G (PKG), which phosphorylates zyxin [29,30]. Phosphorylation of zyxin at serine 142 initiates translocation of zyxin to the nucleus [29]. In the nucleus, zyxin binds a stretch-sensitive promoter sequence, Pypu-box [29]. As a result, mechanical stimulation is translated into gene expression [29]. Our protein–protein interaction analysis indicated that paxillin and vinculin interact with both Myh11 and zyxin. Furthermore, K1256del is predicted to cause misfolding of Myh11 [2]. Taken together, the structural change of Myh11 induced by K1256del may reduce recruitment of paxillin and vinculin, which is required for contractile force development, and binding of zyxin to the focal adhesion complex. Consequentially, zyxin cannot be phosphorylated, blocking its nuclear translocation. Ultimately, transcription activation by zyxin is inhibited upon mechanical stimulation. In the present study, 51 proteins were downregulated in both stretch-stimulated zyxin knockout vascular smooth muscle cells and in Myh11^ΔK/ΔK^ aortas. Thus, we propose that Myh11 K1256 deletion prevents TRPC3/zyxin signaling from upregulating at least those 51 genes, leading to dysregulation of other genes that we identified in this study. To determine whether gene/protein downregulation is primary or secondary to primary expression dysregulation, we need proteomic or transcriptomic profiles of more gene knockout data of proteins that act as transcription factors or activators of transcription factors, such as Yap, Vasp, and Src. Unfortunately, omic datasets of knockouts of those genes in SMCs were not available, so we were unable to assess genes/proteins downregulated by them. In the future, omic analyses of gene knockouts will help identify additional genes that are directly dysregulated in Myh11^ΔK/ΔK^.

It was striking that various proteins associated with connective tissue disease were downregulated in Myh11^ΔK/ΔK^ aortas. For instance, haploinsufficiency or pathogenic variants of Txb are linked to Ehlers–Danlos syndrome [24,25], and pathogenic variants of Tgfb2 and Tgfb receptor 1 and 2 cause Loeys–Dietz syndrome [26,27]. However, in contrast to these connective tissue diseases, Myh11^ΔK/ΔK^ mice and patients with FTAAD caused by MYH11 pathogenic variants do not show syndromic features [2]. Downregulation of genes linked to Ehlers–Danlos and Loeys–Dietz syndromes suggests that the pathogenesis of aortic dissection in Myh11 K1256del mice and patients with FTAAD may show similarities to the pathogenesis of Ehlers–Danlos and Loeys–Dietz syndromes. Downregulation of those genes most likely takes place only in SMCs, since Myh11 expression is limited to VSMCs. This tissue specificity may explain why FTAAD patients, despite having altered expression of genes linked to syndromic diseases, do not present with systemic abnormalities. Furthermore, previous research demonstrated that TGFβ signaling was enhanced in the IVS32+1G>A MYH11 pathogenic variant [31]. Differences in TGFβ signaling may be specific to the location of MYH11 mutations.

In our previous study, 75% of mice with homozygous deletions of K1256 Myh11 died within four days after initiation of angiotensin II treatment. Meta-analysis has shown that the rate of rupture was 7 times higher in aortic dissection patients with patent false lumens than in patients with closed false lumens [32]. Additionally, patients with closed false lumens had better survival during the acute phase [33]. Enrichment analysis showed that Myh11^ΔK/ΔK^ aortas dysregulated coagulation and wound healing. In fact, proteins involved in coagulation, such as platelet factors and thrombomodulin, were downregulated. This may explain why Myh11^ΔK/ΔK^ mice quickly died of aortic rupture in the previous study [2].

The KEGG pathway analysis of this study predicted that aerobic respiration was increased. Potentiation of aerobic respiration may reflect an increased energy demand or impaired energy homeostasis in Myh11^ΔK/ΔK^ aortic tissue. Excessive production of reactive oxygen species (ROS) by aerobic respiration can be reduced by antioxidant networks [34,35]. However, excess ROSs can lead to oxidative stress that harms DNA, proteins, or lipids and even induces cell death by apoptosis or ferroptosis [36,37]. Furthermore, oxidative stress has been linked to the pathophysiology of aortic dissection [38]. Thus, enrichment of aerobic respiration may contribute to the development of aortic dissection.

Even though our previous study indicated calcium transport deficiency in Myh11^ΔK/ΔK^ aortas, expression of key genes that we identified in this study was below the detection level. Likewise, alterations in other pathways may be apparent when a method with higher sensitivity is employed.

We retrieved microarray data of in vivo VSMCs to find dysregulated genes. Using proteomic data of aortas from zyxin knockout mice may provide more accurate expression profiles of zyxin-regulated genes.

In this study, we showed that Myh11^ΔK/ΔK^ aortas are defective in ECM and focal adhesion. We identified one of the primary molecules that links Myh11 structural changes to gene expression among proteins that indirectly interact with Myh11. Zyxin is first recruited to focal adhesion complexes and translocated to the nucleus after being phosphorylated. Myh11 misfolding may ultimately block transcription activation by inhibiting zyxin recruitment and translocation. Then, we identified proteins/genes regulated by zyxin. Those genes may cause secondary dysregulation of other genes. We also discovered downregulation of proteins linked to Loyes–Dietz syndrome and Ehlers–Danlos syndrome. Thus, in the future, comparative omic studies of those syndromes and FTAAD may provide further understanding of FTAAD pathophysiology. In addition to those, we demonstrated an increase in aerobic respiration. Oxidative stress induced by excessive aerobic respiration may contribute to the pathophysiology of FTAAD.

## 4. Materials and Methods

### 4.1. Animals

Previously developed 10- to 12-week-old C57BL/6J mice carrying homozygous K1256 deletions of Myh11 and their wild-type littermates were kept under a 12 h light/dark schedule [2]. We included 5 male mice per group, as in a previous proteomic study [39]. We deeply anesthetized mice before we extracted their aortas. Extracted aortas were immediately frozen in liquid nitrogen. All animal handling procedures in this study complied with the Jichi Medical University Guide for Laboratory Animals and ARRIVE guidelines [40]. The Institutional Animal Care and Concern Committee at Jichi Medical University approved all experimental protocols.

### 4.2. Protein Extraction, Digestion, and Peptide Purification

Proteins were extracted by adding lysis buffer composed of 100 mM Tris-HCl (pH 8.0, 4% SDS, 20 mM NaCl) and 10% acetonitrile (CAN). Cell lysis and protein solubilization were achieved using a sealed ultrasonic homogenizer followed by mixing on a rotator. Protein concentration was measured using the BCA assay, and all samples were normalized to a final concentration of 0.2 μg/μL using the same lysis buffer. For solid-phase-enhanced sample preparation, SP3 beads were prepared by mixing Sera-Mag SpeedBead Carboxylate-Modified Magnetic Particles (hydrophilic and hydrophobic), (Cytiva, Marlborough, MA, USA), at a 1:1 *v/v* ratio. Beads were washed three times with distilled water and adjusted to 8 μg solids/μL. Twenty microliters of SP3 beads were added to each sample, followed by addition of three volumes of propanol. Samples were mixed at room temperature for 20 min to allow protein binding to beads. Bound proteins were washed three times with 80% propanol and once with ethanol, then resuspended in 80 μL of digestion buffer containing 50 mM Tris-HCl (pH 8.0), 10 mM CaCl_2_, and 0.02% lauryl maltose neopentyl glycol (LMNG), as previously described [41]. Digestion was performed by adding 1 μg of Trypsin/Lys-C Mix (Promega, Madison, WI, USA) and incubating at 37 °C for 14 h. After digestion, disulfide bonds were reduced and alkylated by adding TCEP and 2-chloroacetamide to final concentrations of 10 mM and 40 mM, respectively, followed by incubation at 80 °C for 15 min. Digestion was quenched by adding 16 μL of 5% TFA with mixing. Peptides were desalted using GL-Tip SDB spin columns (GL Sciences, Tokyo, Japan), and concentrations were measured using the Fluorometric Peptide Assay (Thermo Fisher Scientific, Waltham, MA, USA). Samples were dried using a centrifugal evaporator and reconstituted in 0.1% TFA with 0.02% LMNG to a final peptide concentration of 200 ng/μL, followed by 10 min of mixing to ensure solubilization.

### 4.3. NanoLC-MS/MS Analysis

Each peptide sample (200 ng) was analyzed using an UltiMate 3000 RSLCnano LC system (Thermo Fisher Scientific) coupled with a Q Exactive HF-X mass spectrometer (Thermo Fisher Scientific). Peptide separation was performed on a C_18_ column (75 μm inner diameter × 120 mm length, Nikkyo Technos, Tokyo, Japan) at 50 °C. Mobile phases comprised 0.1% formic acid in distilled water (Solution A) and 0.1% formic acid in 80% acetonitrile (Solution B). Starting at 6% B, the gradient increased linearly over 32 min to 37%, and then at 38 min, it ramped up to 75% B. That concentration was maintained until the end of the 40 min run. The flow rate was constant at 200 nL/min. Mass spectrometry was performed in positive ESI mode using a data-independent acquisition (DIA) method. Full MS1 scans were acquired at 30,000 resolution with an AGC target of 3 × 10^6^, a maximum injection time of 55 ms, and a scan range of 495–745 *m/z*. DIA MS2 scans were acquired at the same resolution, with an AGC target of 3 × 10^6^, automatic injection time, normalized collision energy of 23%, and 40 isolation windows (6.0 Th) each spanning *m/z* 503.5 to 737.6.

### 4.4. Protein Quantification and Statistical Analysis

Acquired raw data were analyzed using DIA-NN (version 1.9.1). A predicted spectral library was generated using the Mouse UniProtKB/Swiss-Prot database (Proteome ID: UP000000589), 21,709 entries; downloaded 1 April 2024. Library construction was performed with in silico tryptic digestion allowing one missed cleavage, N-terminal methionine excision, and deep-learning-based prediction of spectra, retention times, and ion mobilities. Peptides ranging from 7 to 45 amino acids and carrying charge states of +2 to +4 were included in the analysis. The QuantUMS approach, which was implemented in DIA-NN, was used to quantify proteins. Cross-run normalization was based on retention time. False discovery rate (FDR) thresholds for both precursors and proteins were set at less than 1%. Mass accuracy was limited to 10 ppm for MS1 and MS2. Protein inference was conducted at the gene level, and peptides with shared spectra were excluded from quantification. Before analysis, raw protein expression data were log_2_-transformed. Only proteins with quantification values found in at least 70% of samples from one or more experimental groups were kept after filtering. Imputation parameters were set to a width of 0.3 and a downshift of 1.8, approximating values lower than the detection limit. log_2_ fold change values were computed for each protein. Welch’s *t*-test was used to calculate *p*-values. Adjusted *p*-values were applied using the Benjamini–Hochberg method to adjust for multiple testing.

### 4.5. Fold-Change-Specific Enrichment Analysis

Proteins were first filtered for adjusted *p*-values < 0.05. Using Entrez IDs and their log fold change values as inputs, filtered data were analyzed for enrichment using the FoldGO website (https://webfsgor.sysbio.cytogen.ru/run.html; accessed on 18 March 2025), which has an algorithm to account for fold changes [18].

### 4.6. KEGG Pathway Enrichment Analysis

Proteins whose adjusted *p*-values were less than 0.1 were selected for KEGG pathway enrichment analysis. In R Studio software (version 2024.12.1+563), the enrichKEGG function was used with parameters set to organism = “mmu” (*Mus musculus* and pvalue Cutoff = 0.05.)

### 4.7. Protein–Protein Interaction (PPI) Network and MCODE Clustering

Proteins of interest with adjusted *p*-values < 0.1 were submitted to the STRING database (v11.5) via API to retrieve PPI data specific to *Mus musculus* (taxonomy ID: 10090). Network clustering was performed using the Molecular Complex Detection (MCODE) algorithm implemented in Cytoscape to identify densely connected modules in the PPI network. Parameters used for visualization were as follows: degree cutoff = 2, node score cutoff = 0.2, k-core = 2, and max depth = 100.

### 4.8. Identification of Genes Regulated by Zyxin

Microarray data were retrieved from the Gene Expression Omnibus database (http://www.ncbi.nlm.nih.gov/geo/ accessed one 6 July 2025; accession No. GSE60447) to identify genes upregulated and downregulated in zyxin knockout VSMCs after stretch stimulation [23]. To assess statistical significance, the Kruskal–Wallis test was applied to each gene, comparing expression values between wild-type and zyxin knockout. For comparison of stretched zyxin knockout VSMCs vs. stretched wild-type VSMCs, Dunn’s post hoc test was applied. Log_2_ fold change for each feature was calculated as the difference in mean log_2_ expression between groups. Resulting *p*-values were adjusted for multiple hypothesis testing using the Benjamini–Hochberg procedure to calculate adjusted *p*-values. Genes whose adjusted *p*-values were less than 0.1 were compared to our proteomic data. Proteins/genes downregulated in both Myh11^ΔK/ΔK^ aortas and zyxin knockout VSMCs were selected.

## Figures and Tables

**Table 1:** Top 10 Gene Ontology (GO) terms in *cellular component* enriched among downregulated proteins in Myh11^ΔK/ΔK^ * aortas. GO terms were identified by fold-change-specific enrichment analysis of proteins that were significantly downregulated compared to wild-type aortas.

GO Term	Fold Change
Extracellular region	−0.4–−4.56
Extracellular space	−0.4–−4.56
Extracellular matrix	−0.4–−4.56
Extracellular region part	−0.4–−4.56
Collagen-containing extracellular matrix	−0.4–−4.56
Basement membrane	−0.304–−4.56
Cell projection	−0.304–−0.4
Plasma membrane-bound cell projection	−0.304–−0.4
Nucleosome	−0.178–−0.234
Nuclear nucleosome	−0.178–−0.234

* Homozygous K1256 deletion of Myh11.

**Table 2:** Top 10 Gene Ontology (GO) terms in *molecular function* enriched among downregulated proteins in Myh11^ΔK/ΔK^ * aortas. GO terms were identified by fold-change-specific enrichment analysis of proteins that were significantly downregulated compared to wild-type aortas.

GO Term	Fold-Change
Extracellular matrix structural constituent conferring tensile strength	−0.623–−4.56
Serine-type endopeptidase activity	−0.4–−4.56
Enzyme inhibitor activity	−0.4–−4.56
Endopeptidase inhibitor activity	−0.4–−4.56
Extracellular matrix structural constituent	−0.4–−4.56
Glycosaminoglycan binding	−0.4–−4.56
Heparin binding	−0.4–−4.56
Serine-type peptidase activity	−0.4–−4.56
Serine hydrolase activity	−0.4–−4.56
Peptidase inhibitor activity	−0.4–−4.56

* Homozygous K1256 deletion of Myh11.

**Table 3:** Top 12 Gene Ontology (GO) terms in *biological process* enriched among downregulated proteins in Myh11^ΔK/ΔK^ * aortas. GO terms were identified by fold-change-specific enrichment analysis of proteins that were significantly downregulated compared to wild-type aortas.

GO Term	Fold Change
Blood coagulation	−0.623–−4.56
Hemostasis	−0.623–−4.56
Regulation of blood coagulation	−0.623–−4.56
Negative regulation of blood coagulation	−0.623–−4.56
Coagulation	−0.623–−4.56
Regulation of coagulation	−0.623–−4.56
Negative regulation of coagulation	−0.623–−4.56
Regulation of body fluid levels	−0.623–−4.56
Negative regulation of wound healing	−0.623–−4.56
Regulation of hemostasis	−0.623–−4.56
Negative regulation of hemostasis	−0.623–−4.56
Negative regulation of response to wounding	−0.623–−4.56

* Homozygous K1256 deletion of Myh11.

**Table 4:** Top 12 Gene Ontology (GO) terms enriched among upregulated proteins in Myh11^ΔK/ΔK^ * aortas. GO terms were identified by fold-change-specific enrichment analysis of proteins that were significantly upregulated compared to wild-type aortas.

GO Term	Fold-Change
Organellar ribosome	0.358–4.43
Organellar large ribosomal subunit	0.358–4.43
Mitochondrial respiratory chain	0.358–4.43
Mitochondrial matrix	0.358–4.43
Mitochondrial ribosome	0.358–4.43
Mitochondrial large ribosomal subunit	0.358–4.43
Mitochondrial part	0.358–4.43
Mitochondrial membrane part	0.358–4.43
Respiratory chain	0.358–4.43
Inner mitochondrial membrane protein complex	0.358–4.43
Respiratory chain complex	0.358–4.43
Oxidoreductase complex	0.358–4.43

* Homozygous K1256 deletion of Myh11.

**Table 5:** Enriched pathways common to both downregulated genes and proteins identified by fold-change-specific enrichment analysis. Pathway enrichment analysis was performed separately on transcriptomic and proteomic datasets using fold-change-specific criteria. This table lists pathways that were significantly enriched among downregulated genes and proteins in Myh11^ΔK/ΔK^ * aortas and that were common to both datasets, indicating consistent pathway-level regulation across the transcriptome and proteome.

GO ** Term	Fold-Change
Cell projection	−0.304–−4.56
Inorganic molecular entity transmembrane transporter activity	−0.304–−0.623
Plasma membrane-bound cell projection	−0.304–−0.4
Response to wounding	−0.304–−0.4
Organelle organization	−0.094–−0.4
Intracellular	−0.094–−0.4
Nucleus	−0.094–−0.4
Cytoplasm	−0.094–−0.4
Organelle	−0.094–−0.4
Membrane-bound organelle	−0.094–−0.4
Intracellular organelle	−0.094–−0.4
Intracellular membrane-bound organelle	−0.094–−0.4
Intracellular part	−0.094–−0.4
Nuclear part	−0.094–−0.4
Cytoplasmic part	−0.094–−0.4
Intracellular organelle part	−0.094–−0.4
Catalytic complex	−0.094–−0.304
Organic cyclic compound binding	−0.094–−0.304
Heterocyclic compound binding	−0.094–−0.178

* Homozygous K1256 deletion of Myh11. ** Gene Ontology.

## Data Availability

The original contributions presented in this study are included in the article/Supplementary Material. Further inquiries can be directed to the corresponding author.

## Supplementary Materials

The following supporting information can be downloaded at https://www.mdpi.com/article/10.3390/ijms26167853/s1.
