# Supplementary material for: Loss of Myh11 K1256 Dysregulates the Extracellular Matrix and Focal Adhesion by Inhibiting Zyxin-Activated Transcription
Source: Int J Mol Sci. 2025 Aug 14;26(16):7853. doi: 10.3390/ijms26167853 (PMC12386218; doi:10.3390/ijms26167853)
Supplement: Supplementary file 1 [file ijms-26-07853-s001.zip › ijms-3700227-supplementary.pdf]

Supplementary information

Supplementary Table S1. The table shows proteins and their log2 fold change that were enriched in the ECM-receptor interaction.

Protein	Log2 Fold Change
Agrn	-0.72
Cd44	-0.40
Chad	-0.47
Col4a5	-0.85
Col6a1	-0.78
Col6a2	-0.73
Col6a3	-0.70
Dag1	-0.26
Fn1	-0.50
Gp1ba	-1.85
Gp1bb	-1.88
Gp5	-1.92
Hspg2	-0.55
Itga2b	-1.31
Itga3	-0.39
Itga5	-0.56
Itga7	0.21
Itgav	-0.38
Itgb1	-0.25
Itgb3	-0.29
Itgb5	-0.70
Lama2	-0.45

Lama4	-0.40
Lamb2	-0.45
Sdc4	-0.42
Thbs3	-0.71
Tnc	-0.40
Vtn	-1.07
Vwf	-0.48
Lamc3	-0.34
Tnxb	-1.06
Col4a6	-0.96
Itga1	0.30
Npnt	-0.54
Lamc1	-0.36
Itga8	-0.35
Col6a6	-0.68
Col6a5	-0.42

Supplementary Table S2. The table shows proteins and their log2 fold change that were enriched in pyruvate metabolism.

**Protein**	**Log2 Fold Change**
Adh5	0.27
Aldh3a2	0.15
Ldha	0.16
Ldhb	0.44
Me1	0.18
Mdh2	0.50

Mdh1	0.27
Pkm	0.23
Acyp1	0.48
Acss1	0.96
Pck2	0.13
Acyp2	0.37
Grhpr	0.51
Glo1	0.20
Acat1	0.28
Acat2	0.38
Aldh7a1	0.11

Supplementary Table S3. The table shows proteins and their log2 fold change that were enriched in hypertrophic cardiomyopathy.

**Protein**	**Log2 Fold Change**
Ace	-0.83
Atp2a2	0.11
Cacna2d1	-0.43
Dag1	-0.26
Des	-0.17
Dmd	-0.13
Dtna	-0.31
Emd	-0.14
Itga2b	-1.31
Itga3	-0.39
Itga5	-0.56

Itga7	0.21
Itgav	-0.38
Itgb1	-0.25
Itgb3	-0.29
Itgb5	-0.70
Lama2	-0.45
Lmna	-0.11
Mybpc3	4.11
Prkab1	-0.11
Ryr2	0.23
Sgca	-0.46
Sgce	-0.35
Sntb2	-0.36
Tgfb1	-0.61
Tgfb2	-0.31
Tnni3	3.87
Tpm1	0.21
Tpm2	0.39
Sgcb	-0.24
Sgcd	-0.22
Sgcg	-0.38
Tpm3	0.55
Prkaa2	0.33
Itga1	0.30
Slc8a2	-0.16
Itga8	-0.35

Tpm4	-0.22

Supplementary Table S4. The table shows proteins and their log2 fold change that were enriched in arrhythmogenic right ventricular cardiomyopathy.

**Protein**	**Log2 Fold Change**
Atp2a2	0.11
Cacna2d1	-0.43
Ctnna1	0.25
Ctnnb1	0.45
Dag1	-0.26
Des	-0.17
Dmd	-0.13
Dtna	-0.31
Emd	-0.14
Itga2b	-1.31
Itga3	-0.39
Itga5	-0.56
Itga7	0.21
Itgav	-0.38
Itgb1	-0.25
Itgb3	-0.29
Itgb5	-0.70
Jup	0.34
Lama2	-0.45
Lmna	-0.11
Ryr2	0.23

Sgca	-0.46
Sgce	-0.35
Sntb2	-0.36
Sgcb	-0.24
Sgcd	-0.22
Sgcg	-0.38
Dsp	-0.33
Itga1	0.30
Slc8a2	-0.16
Ctnna3	0.49
Itga8	-0.35

Supplementary Table S5. The table shows proteins and their log2 fold change that were enriched in dilated cardiomyopathy.

**Protein**	**Log2 Fold Change**
Adcy7	-0.26
Atp2a2	0.11
Cacna2d1	-0.43
Dag1	-0.26
Des	-0.17
Dmd	-0.13
Dtna	-0.31
Emd	-0.14
Itga2b	-1.31
Itga3	-0.39
Itga5	-0.56

Itga7	0.21
Itgav	-0.38
Itgb1	-0.25
Itgb3	-0.29
Itgb5	-0.70
Lama2	-0.45
Lmna	-0.11
Mybpc3	4.11
Prkaca	0.15
Prkacb	0.12
Ryr2	0.23
Sgca	-0.46
Sgce	-0.35
Sntb2	-0.36
Tgfb1	-0.61
Tgfb2	-0.31
Tnni3	3.87
Tpm1	0.21
Tpm2	0.39
Sgcb	-0.24
Sgcd	-0.22
Sgcg	-0.38
Tpm3	0.55
Itga1	0.30
Slc8a2	-0.16
Itga8	-0.35

Tpm4	-0.22

Supplementary Table S6. The table shows proteins and their log2 fold change that were enriched in the cytoskeleton in muscle cells.

**Protein**	**Log2 Fold Change**
Agrn	-0.72
Atp1b1	-0.45
Atp1b2	0.16
Atp1b3	-0.11
Bgn	-0.41
Capzb	0.15
Col4a5	-0.85
Col6a1	-0.78
Col6a2	-0.73
Col6a3	-0.70
Vcan	-0.46
Csrp1	0.36
Dag1	-0.26
Dcn	-0.29
Des	-0.17
Diaph1	-0.35
Dmd	-0.13
Dtna	-0.31
Emd	-0.14
Eno1	0.33
Eno2	0.34

Fbln2	-0.27
Fhl1	-0.17
Fhl3	-0.82
Fn1	-0.50
Hspg2	-0.55
Itga2b	-1.31
Itga3	-0.39
Itga5	-0.56
Itga7	0.21
Itgav	-0.38
Itgb1	-0.25
Itgb3	-0.29
Itgb5	-0.70
Jup	0.34
Lama2	-0.45
Lmna	-0.11
Lmnb2	-0.20
Mybpc3	4.11
Myh11	0.42
Myh9	-0.28
Myl4	1.11
Myom1	0.45
Plec	-0.25
Sgca	-0.46
Sgce	-0.35
Sntb2	-0.36

Sdc4	-0.42
Thbs3	-0.71
Tln1	-0.10
Tmod1	0.32
Tnni3	3.87
Tpm1	0.21
Tpm2	0.39
Vcl	-0.34
Xirp1	0.31
Zyx	-0.68
Sgcb	-0.24
Sgcd	-0.22
Sgcg	-0.38
Ldb3	0.24
Pdlim4	-0.31
Myoz1	0.76
Tpm3	0.55
Syne1	-0.10
Flnc	-0.60
Tmem43	-0.44
Daam2	0.52
Sun1	-0.25
Myh10	0.14
Lmod1	0.27
Col4a6	-0.96
Myl9	0.36

Dsp	-0.33
Itga1	0.30
Syne3	-0.32
Pdlim2	-0.42
Sun2	-0.18
Itga8	-0.35
Col6a6	-0.68
Syne2	-0.28
Tpm4	-0.22
Eno1b	0.33
Col6a5	-0.42

Supplementary Table S7. The table shows proteins and their log2 fold change that were enriched in focal adhesion.

**Protein**	**Log2 Fold Change**
Akt1	0.17
Akt2	0.15
Capn2	0.08
Ctnnb1	0.45
Chad	-0.47
Col4a5	-0.85
Col6a1	-0.78
Col6a2	-0.73
Col6a3	-0.70
Bcar1	-0.39
Crkl	-0.14

Diaph1	-0.35
Egfr	-0.20
Flt1	-0.39
Fn1	-0.50
Ilk	-0.21
Itga2b	-1.31
Itga3	-0.39
Itga5	-0.56
Itga7	0.21
Itgav	-0.38
Itgb1	-0.25
Itgb3	-0.29
Itgb5	-0.70
Lama2	-0.45
Lama4	-0.40
Lamb2	-0.45
Ppp1r12a	0.20
Pdgfrb	-0.12
Pik3ca	-0.08
Pip5k1a	-0.16
Prkca	-0.23
Prkcb	-0.42
Ppp1ca	-0.11
Ppp1cb	0.27
Ppp1cc	0.11
Pxn	-0.38

Rock1	0.60
Rock2	0.16
Src	-0.32
Thbs3	-0.71
Tln1	-0.10
Tnc	-0.40
Vasp	-0.55
Vcl	-0.34
Vtn	-1.07
Vwf	-0.48
Zyx	-0.68
Lamc3	-0.34
Vav3	0.56
Parva	-0.18
Myl12b	0.29
Pak4	-0.20
Pdgfd	-0.27
Pik3cb	-0.14
Tnxb	-1.06
Col4a6	-0.96
Myl9	0.36
Mylk	0.36
Rapgef1	0.16
Itga1	0.30
Actn1	0.26
Parvb	-2.28

Rap1b	-0.14
Pak2	-0.09
Lamc1	-0.36
Mylk2	-0.30
Ppp1r12c	0.37
Itga8	-0.35
Col6a6	-0.68
Ppp1r12b	0.35
Col6a5	-0.42

Supplementary Table S8. The table shows proteins and their log2 fold change that were enriched in oxidative phosphorylation.

**Protein**	**Log2 Fold Change**
Atp5f1a	0.27
Atp5f1b	0.20
Atp5f1c	0.31
Atp5pb	0.65
Atp5pf	1.10
Atp5me	0.63
Atp6v1a	0.11
Atp6v1b2	0.17
Atp6v0c	-0.68
Cox4i1	0.64
Cox5b	0.57
Cycs	0.49
Cytb	0.46

Ndufa4	0.44
Ndufs4	0.35
Atp5mg	0.33
Atp5po	0.41
Atp5mf	0.46
Atp5f1d	0.30
Ndufb5	0.80
Sdhc	0.33
Ndufb9	0.40
Atp6v1g1	0.26
Atp6v1c1	0.15
Ndufa12	0.42
Ndufa7	0.57
Cyc1	0.41
Ndufb3	0.59
Ndufb7	0.58
Ndufb8	0.66
Ndufa10	0.45
Uqcrb	0.89
Ndufb4	0.80
Ndufc2	0.49
Ndufa5	0.67
Ndufa8	0.46
Ndufa11	0.38
Atp5pd	0.30
Ndufv2	0.69

Ppa2	0.20
Ndufs7	0.46
Lhpp	0.30
Ndufv3	0.78
Cox4i2	1.27
Atp6v1h	0.11
Cox6b1	0.63
Ndufs2	0.33
Ndufs1	0.37
Ndufs6	0.55
Ndufs6b	0.55

Supplementary Table S9. The table shows proteins and their log2 fold change that were enriched in glycolysis/gluconeogenesis.

**Protein**	**Log2 Fold Change**
Adh5	0.27
Aldh3a2	0.15
Aldoa	0.31
Bpgm	-0.67
Eno1	0.33
Eno2	0.34
Gpi1	0.30
Ldha	0.16
Ldhb	0.44
Minpp1	-0.33
Pfkl	0.09

Pfkm	0.24
Pgam1	0.13
Pgk1	0.27
Pkm	0.23
Tpi1	0.19
Pgam2	1.09
Acss1	0.96
Pck2	0.13
Aldh7a1	0.11
Galm	0.15
Eno1b	0.33

Supplementary Table S10. The table shows proteins and their log_2 fold change that were enriched in diabetic cardiomyopathy.

**Protein**	**Log2 Fold Change**
Ace	-0.83
Akt1	0.17
Akt2	0.15
Slc25a4	0.54
Slc25a5	0.56
Atp2a2	0.11
Atp5f1a	0.27
Atp5f1b	0.20
Atp5f1c	0.31
Atp5pb	0.65
Atp5pf	1.10

Cox4i1	0.64
Cox5b	0.57
Cyba	-0.28
Gys1	0.38
Smad2	-0.24
Mmp2	-0.40
Cytb	0.46
Ndufa4	0.44
Ndufs4	0.35
Pik3ca	-0.08
Prkca	-0.23
Prkcb	-0.42
Prkcd	0.45
Plcb1	-0.13
Plcb4	-0.14
Ppp1ca	-0.11
Ppp1cb	0.27
Ppp1cc	0.11
Ryr2	0.23
Slc2a1	-0.34
Tgfb1	-0.61
Tgfb2	-0.31
Tgfbr1	-0.41
Tnni3	3.87
Vdac1	0.31
Vdac2	0.28

Vdac3	0.19
Atp5po	0.41
Atp5f1d	0.30
Ndufb5	0.80
Sdhc	0.33
Ndufb9	0.40
Ndufa12	0.42
Ndufa7	0.57
Cyc1	0.41
Ndufb3	0.59
Ndufb7	0.58
Ndufb8	0.66
Ndufa10	0.45
Uqcrb	0.89
Ndufb4	0.80
Ndufc2	0.49
Ndufa5	0.67
Ndufa8	0.46
Ndufa11	0.38
Atp5pd	0.30
Ndufv2	0.69
Pik3cb	-0.14
Ndufs7	0.46
Ndufv3	0.78
Cox4i2	1.27
Cox6b1	0.63

Ptpa	0.32
Ndufs2	0.33
Ndufs1	0.37
Pdk3	0.10
Ndufs6	0.55
Ndufs6b	0.55

Supplementary Table S11. The table shows proteins and their log_2 fold change that were enriched in inositol phosphate metabolism.

**Protein**	**Log2 Fold Change**
Inppl1	-0.29
Minpp1	-0.33
Pik3ca	-0.08
Pip4k2a	0.51
Pip5k1a	-0.16
Plcb1	-0.13
Plcb4	-0.14
Inpp5k	0.24
Synj2	-0.10
Tpi1	0.19
Mtmr7	-1.43
Impa1	0.28
Plce1	0.96
Pik3cb	-0.14
Sacm1l	0.17
Inpp5f	-0.26

Pi4kb	-0.20
Pip4k2b	-0.19
Pip4k2c	0.29
Inpp5a	0.15
Itpk1	0.38
Inpp4a	0.19

Supplementary Table S12. The table shows proteins and their $\log_2$ fold change that were enriched in the regulation of actin cytoskeleton.

**Protein**	**Log2 Fold Change**
Akt1	0.17
Akt2	0.15
C9	-0.46
Bcar1	-0.39
Crkl	-0.14
Diaph1	-0.35
Egfr	-0.20
F2	-0.65
Fgf1	0.32
Fgf2	0.17
Fgfr1	0.20
Fn1	-0.50
Gng12	1.05
Itga2b	-1.31
Itga3	-0.39
Itga5	-0.56

Itga7	0.21
Itgav	-0.38
Itgb1	-0.25
Itgb3	-0.29
Itgb5	-0.70
Msn	-0.12
Myh11	0.42
Myh9	-0.28
Ppp1r12a	0.20
Pdgfrb	-0.12
Pik3ca	-0.08
Pip4k2a	0.51
Pip5k1a	-0.16
Ppp1ca	-0.11
Ppp1cb	0.27
Ppp1cc	0.11
Pxn	-0.38
Rdx	0.12
Rock1	0.60
Rock2	0.16
Slc9a1	-0.47
Src	-0.32
Vcl	-0.34
Map2k2	0.13
Arhgef7	0.12
Arpc1a	0.28

Vav3	0.56
Myl12b	0.29
Arpc4	0.24
C8g	-0.65
Pak4	-0.20
Pdgfd	-0.27
Wasl	-0.47
Arpc5l	0.29
Pik3cb	-0.14
Myh10	0.14
Myl9	0.36
Mylk	0.36
Pip4k2b	-0.19
Itga1	0.30
Actn1	0.26
C7	-0.40
C8b	-0.53
Pip4k2c	0.29
Git1	-0.23
Pak2	-0.09
Mylk2	-0.30
C8a	-0.52
Ppp1r12c	0.37
Itga8	-0.35
Actr3b	0.45
Ssh3	-0.16

Abi2	0.24
Ppp1r12b	0.35

Supplementary Table S13. The table shows proteins and their log_2 fold change that were enriched in nucleotide metabolism.

**Protein**	**Log2 Fold Change**
Ada	0.22
Adk	-0.21
Adsl	0.15
Adss1	0.58
Ak4	1.34
Entpd1	-0.11
Hprt1	0.20
Nme1	0.25
Nme2	0.32
Enpp1	0.14
Pnp	0.30
Dtymk	0.55
Uck1	0.39
Ctps2	-0.13
Ak3	0.66
Gmpr	0.65
Cmpk1	0.19
Nt5c3b	0.27
Hddc2	0.32
Nudt16	0.28

Nt5c2	0.22
Nme3	0.24
Gmpr2	0.19
Nme7	0.13
Enpp3	-0.27

Supplementary Table S14. The table shows proteins and their log_2 fold change that were enriched in cardiac muscle contraction.

Protein	Log2 Fold Change
Atp1b1	-0.45
Atp1b2	0.16
Atp1b3	-0.11
Atp2a2	0.11
Cacna2d1	-0.43
Casq2	2.30
Cox4i1	0.64
Cox5b	0.57
Hrc	0.78
Cytb	0.46
Myl4	1.11
Ryr2	0.23
Slc9a1	-0.47
Tnni3	3.87
Tpm1	0.21
Tpm2	0.39
Tpm3	0.55

Asph	-0.24
Cyc1	0.41
Uqcrb	0.89
Cox4i2	1.27
Cox6b1	0.63
Slc8a2	-0.16
Slc9a6	-0.31
Tpm4	-0.22

Supplementary Table S15. The table shows proteins and their log_2 fold change that were enriched in thermogenesis.

**Protein**	**Log2 Fold Change**
Adcy7	-0.26
Atp5f1a	0.27
Atp5f1b	0.20
Atp5f1c	0.31
Atp5pb	0.65
Atp5pf	1.10
Atp5me	0.63
Cox4i1	0.64
Cox5b	0.57
Cpt1a	0.19
Creb1	-0.37
Fgfr1	0.20
Cytb	0.46
Ndufa4	0.44

Ndufs4	0.35
Prkaca	0.15
Prkacb	0.12
Prkab1	-0.11
Prkg1	0.32
Smarca4	-0.16
Atp5mg	0.33
Atp5po	0.41
Sirt6	1.07
Actl6a	-0.10
Atp5mf	0.46
Tsc1	-0.57
Atp5f1d	0.30
Ndufb5	0.80
Sdhc	0.33
Ndufb9	0.40
Ndufa12	0.42
Ndufa7	0.57
Cyc1	0.41
Ndufb3	0.59
Ndufaf3	0.42
Ndufb7	0.58
Smarcd3	-0.08
Ndufb8	0.66
Ndufa10	0.45
Uqcrb	0.89

Smarcc2	-0.08
Ndufb4	0.80
Ndufc2	0.49
Ndufa5	0.67
Ndufa8	0.46
Ndufaf4	0.30
Ndufaf5	0.87
Ndufa11	0.38
Atp5pd	0.30
Ndufv2	0.69
Ndufaf7	0.31
Ndufs7	0.46
Cpt1c	-0.63
Ndufv3	0.78
Cox4i2	1.27
Prkaa2	0.33
Cox6b1	0.63
Rps6ka3	-0.18
Ndufs2	0.33
Ndufs1	0.37
Ndufs6	0.55
Ndufs6b	0.55

Supplementary Table S16. The table shows proteins and their log_2 fold change that were enriched in the thyroid hormone signaling pathway.

**Protein**	**Log2 Fold Change**

Akt1	0.17
Akt2	0.15
Atp1b1	-0.45
Atp1b2	0.16
Atp1b3	-0.11
Atp2a2	0.11
Ctnnb1	0.45
Crebbp	-0.21
Esr1	-1.60
Itgav	-0.38
Itgb3	-0.29
Pfkl	0.09
Pfkm	0.24
Pik3ca	-0.08
Prkaca	0.15
Prkacb	0.12
Prkca	-0.23
Prkcb	-0.42
Plcb1	-0.13
Plcb4	-0.14
Med1	-0.23
Ncor1	-0.12
Sin3a	-0.15
Slc2a1	-0.34
Slc9a1	-0.47
Src	-0.32

Stat1	0.32
Map2k2	0.13
Plce1	0.96
Pik3cb	-0.14
Hdac1	-0.17

Supplementary Table S17. The table shows proteins and their log_2 fold change that were enriched in purine metabolism.

Protein	Log2 Fold Change
Ada	0.22
Adcy7	-0.26
Adk	-0.21
Adsl	0.15
Adss1	0.58
Ak4	1.34
Entpd1	-0.11
Hprt1	0.20
Nme1	0.25
Nme2	0.32
Pde1c	1.51
Enpp1	0.14
Pnp	0.30
Papss1	-0.21
Papss2	-0.33
Nudt5	0.32
Gucy1b1	-0.17

Pde3a	-0.36
Ak3	0.66
Gucy1a1	-0.18
Gmpr	0.65
Adprm	-1.99
Paics	0.23
Hddc2	0.32
Nudt16	0.28
Nt5c2	0.22
Nme3	0.24
Gmpr2	0.19
Prps2	0.15
Nme7	0.13
Enpp3	-0.27
Pfas	-0.27
Pde4d	-0.47
Pde5a	-0.15

Supplementary Table S18. The table shows proteins and their log_2 fold change that were enriched in non-alcoholic fatty liver disease.

**Protein**	**Log2 Fold Change**
Akt1	0.17
Akt2	0.15
Bax	0.43
Cox4i1	0.64
Cox5b	0.57

Cycs	0.49
Eif2s1	0.24
Cytb	0.46
Ndufa4	0.44
Ndufs4	0.35
Pik3ca	-0.08
Prkab1	-0.11
Tgfb1	-0.61
Ndufb5	0.80
Sdhc	0.33
Ndufb9	0.40
Ndufa12	0.42
Ndufa7	0.57
Cyc1	0.41
Ndufb3	0.59
Ndufb7	0.58
Ndufb8	0.66
Ndufa10	0.45
Uqcrb	0.89
Ndufb4	0.80
Ndufc2	0.49
Ndufa5	0.67
Ndufa8	0.46
Ndufa11	0.38
Ndufv2	0.69
Pik3cb	-0.14

Ndufs7	0.46
Ndufv3	0.78
Cox4i2	1.27
Prkaa2	0.33
Cox6b1	0.63
Ndufs2	0.33
Ndufs1	0.37
Irs2	0.96
Ndufs6	0.55
Ndufs6b	0.55

Supplementary Table S19. The table shows proteins and their log_2 fold change that were enriched in protein processing in the endoplasmic reticulum.

**Protein**	**Log2 Fold Change**
Bax	0.43
Hyou1	-0.24
Pdia4	-0.15
Calr	-0.24
Canx	-0.23
Capn2	0.08
Casp12	-0.15
Cryab	0.27
Ddost	-0.11
Dnajc1	-0.22
Eif2s1	0.24
Ganab	-0.13

Pdia3	-0.19
Hspa5	-0.21
Hspa1l	-0.86
Dnaja1	-0.20
Hspa1b	-0.94
Hspa4l	-0.15
Plaa	0.12
Rad23b	-0.16
Rpn2	-0.15
Sec23a	-0.14
Sel1l	-0.19
Hsp90b1	-0.20
Ufd1	0.14
Cul1	0.11
Sec23b	-0.24
Ero1a	-0.13
Rbx1	0.61
Dnajc10	-0.34
Lman2	-0.19
Ube2g1	-0.29
Erp29	-0.22
Stt3b	-0.11
Sec31a	-0.13
Sec24d	-0.23
Tusc3	-0.34
Dnajb1	-0.29

Txndc5	-0.17
Sec13	-0.14
Bag2	0.20
Man1b1	-0.17
Uggt1	-0.18

Supplementary Table S20. The table shows proteins and their log_2 fold change that were enriched in endocytosis.

**Protein**	**Log2 Fold Change**
Ap2a1	-0.20
Ap2m1	-0.11
Capzb	0.15
Asap1	-0.33
Egfr	-0.20
Epn1	-0.16
Epn2	-0.38
Eps15l1	-0.13
Hgs	-0.15
Hspa1l	-0.86
Hspa1b	-0.94
Ldlr	-0.37
Smad2	-0.24
Pip5k1a	-0.16
Pml	0.14
Cyth3	-0.18
Rab11b	0.13

Rab5b	0.17
Rab7	0.13
Sh3gl1	-0.13
Src	-0.32
Tgfbr1	-0.41
Snf8	0.16
Vps25	0.47
Rab11fip5	-0.24
Vps37a	-0.52
Rabep1	-0.16
Snx12	0.18
Snx1	0.10
Arpc1a	0.28
Ehd3	-0.24
Vta1	0.28
Chmp3	0.22
Vps28	0.31
Arpc4	0.24
Chmp2a	0.17
Vps26b	0.15
Snx4	-0.13
Snx5	0.09
Smap2	-0.20
Vps36	0.27
Ap2b1	-0.18
Wasl	-0.47

Mvb12a	0.27
Arpc5l	0.29
Rab35	0.29
Ehd4	0.13
Ldlrap1	-0.34
Grk2	0.16
Vps4a	0.15
Asap2	-0.33
Wipf1	-0.35
Eea1	-0.18
Git1	-0.23
Sh3glb2	0.21
Spart	0.15
Iqsec1	-0.22
Ap2s1	-0.19
Actr3b	0.45
Ehd2	-0.24
Zfyve27	0.41

Supplementary Table S21. The table shows proteins and their log_2 fold change that were enriched in the extracellular region.

**Protein**	**Log2 Fold Change**
Abi3bp	-0.88
Acan	-0.60
Ace	-0.83
Adamts1	-1.58

Adamtsl4	-0.48
Adamtsl5	-0.46
Aebp1	-0.79
Agrn	-0.72
Ahsg	-0.49
Ang	-0.44
Angpt2	-1.65
Angptl2	-0.49
Apoa1	-0.54
Apoa4	-0.65
App	-1.03
Atrn	-0.51
Azgp1	-0.42
Bche	-0.65
Bgn	-0.41
Bmp1	-1.47
Bmp3	-1.74
C2	-0.56
C3	-0.48
C8a	-0.52
C8b	-0.53
C8g	-0.65
C9	-0.46
Ccdc3	-0.93
Ccdc80	-0.70
Ccl9	-0.44

Ccn1	-0.69
Ccn2	-0.68
Cd109	-1.12
Cd9	-1.24
Ces2e	-0.94
Cfb	-0.72
Cfh	-0.73
Cfi	-0.64
Cfp	-1.27
Chad	-0.47
Clec11a	-0.44
Clstn1	-0.70
Clu	-0.73
Col15a1	-0.82
Col18a1	-0.66
Col4a6	-0.96
Col6a1	-0.78
Col6a2	-0.73
Col6a3	-0.70
Col6a6	-0.68
Cpn1	-0.76
Crp	-1.00
Cyb5d2	-0.63
Cyp4a12b	-0.67
Dkk3	-0.51
Ecm1	-0.72

Ecm2	-0.52
Efemp2	-0.43
Efna5	-0.42
F10	-0.64
F13a1	-0.44
F13b	-1.27
F2	-0.65
F5	-2.89
Fetub	-0.48
Fgl2	-0.41
Fibin	-0.98
Fstl1	-0.46
Gas6	-0.61
Gc	-0.52
Gp1ba	-1.85
Gpc1	-0.43
Gpc4	-0.44
Gpc6	-0.64
Gpx3	-0.57
Hspg2	-0.55
Htra1	-0.94
Htra3	-0.90
Htra4	-1.38
Igfbp5	-0.49
Igfbp6	-0.60
Igfbp7	-0.65

Inhca	-0.50
Itgbl1	-0.64
Itih1	-0.60
Itih4	-0.69
Kera	-0.73
Lama2	-0.45
Lama4	-0.40
Lamb2	-0.45
Lamc1	-0.36
Lgals3	-0.49
Loxl1	-0.59
Loxl2	-0.79
Loxl3	-0.69
Lrrc17	-1.24
Ltbp3	-0.45
Ltbp4	-0.56
Mamdc2	-1.44
Masp1	-1.47
Masp2	-0.82
Matn2	-0.79
Mbl1	-0.96
Mbl2	-0.64
Mfap5	-0.50
Mfge8	-0.75
Mgp	-0.80
Mmp17	-0.81

Mmp2	-0.40
Mmrn1	-1.31
Mst1	-0.48
Mug1	-0.71
Npnt	-0.54
Ntn1	-1.24
Pam	-0.52
Pamr1	-0.56
Pcif1	-0.45
Pcolce2	-0.95
Pdgfrl	-0.94
Pf4	-1.23
Pkd1	-0.40
Plg	-0.73
Pltp	-0.43
Plxdc2	-0.53
Postn	-0.98
Ppp1r13l	-0.55
Prelp	-0.40
Pros1	-1.00
Prss23	-0.46
Qpct	-0.97
Qsox1	-0.60
Rbp4	-0.43
Rnase4	-0.65
Scube3	-0.95

Sdc4	-0.42
Sdcbp	-0.63
Selenop	-1.52
Selp	-1.57
Sema3c	-0.88
Sema3d	-4.29
Sema3f	-0.55
Serpina1a	-0.57
Serpina1b	-0.53
Serpina1d	-0.58
Serpina3k	-0.47
Serpina3n	-0.46
Serpinb8	-0.46
Serpinc1	-0.42
Serpind1	-0.75
Serpine1	-1.08
Serpine2	-0.97
Serpinf2	-0.98
Serpini1	-0.79
Sfrp1	-0.42
Sfrp2	-0.53
Smoc1	-0.95
Smpdl3b	-0.45
Sod3	-0.84
Sorl1	-0.41
Spon1	-0.47

Spon2	-0.58
Spp2	-1.31
Srpx2	-0.64
Sulf1	-0.54
Sulf2	-0.89
Tgfb1	-0.61
Tgfbi	-0.59
Thbd	-0.64
Thbs3	-0.71
Thsd4	-0.66
Timp2	-0.41
Timp3	-0.67
Tnxb	-1.06
Trf	-0.44
Ttr	-0.47
Vcan	-0.46
Vtn	-1.07
Vwf	-0.48

Supplementary Table S22. The table shows proteins and their $\log_2$ fold change that were enriched in extracellular spaces.

**Protein**	**Log2 Fold Change**
Acan	-0.60
Ace	-0.83
Adamtsl4	-0.48
Aebp1	-0.79

Agrn	-0.72
Ahsg	-0.49
Ang	-0.44
Angpt2	-1.65
Angptl2	-0.49
Apoa1	-0.54
Apoa4	-0.65
App	-1.03
Atrn	-0.51
Azgp1	-0.42
Bche	-0.65
Bgn	-0.41
Bmp1	-1.47
Bmp3	-1.74
C2	-0.56
C3	-0.48
C8a	-0.52
C8b	-0.53
C8g	-0.65
C9	-0.46
Ccdc80	-0.70
Ccl9	-0.44
Ccn1	-0.69
Ccn2	-0.68
Cd109	-1.12
Cd9	-1.24

Ces2e	-0.94
Cfb	-0.72
Cfh	-0.73
Cfi	-0.64
Chad	-0.47
Clec11a	-0.44
Clstn1	-0.70
Clu	-0.73
Col15a1	-0.82
Col18a1	-0.66
Col4a6	-0.96
Col6a1	-0.78
Col6a2	-0.73
Col6a3	-0.70
Cpn1	-0.76
Crp	-1.00
Cyp4a12b	-0.67
Dkk3	-0.51
Ecm1	-0.72
Efemp2	-0.43
F10	-0.64
F2	-0.65
F5	-2.89
Fetub	-0.48
Fstl1	-0.46
Gas6	-0.61

Gc	-0.52
Gp1ba	-1.85
Gpc4	-0.44
Gpx3	-0.57
Hspg2	-0.55
Htra1	-0.94
Igfbp5	-0.49
Igfbp6	-0.60
Igfbp7	-0.65
Inhca	-0.50
Itgbl1	-0.64
Lama2	-0.45
Lama4	-0.40
Lamb2	-0.45
Lamc1	-0.36
Lgals3	-0.49
Loxl1	-0.59
Loxl2	-0.79
Loxl3	-0.69
Lrrc17	-1.24
Ltbp4	-0.56
Masp1	-1.47
Masp2	-0.82
Matn2	-0.79
Mbl1	-0.96
Mbl2	-0.64

Mfap5	-0.50
Mfge8	-0.75
Mgp	-0.80
Mmp2	-0.40
Mst1	-0.48
Mug1	-0.71
Npnt	-0.54
Pam	-0.52
Pamr1	-0.56
Pf4	-1.23
Pkd1	-0.40
Plg	-0.73
Pltp	-0.43
Postn	-0.98
Prelp	-0.40
Pros1	-1.00
Prss23	-0.46
Qsox1	-0.60
Rbp4	-0.43
Rnase4	-0.65
Scube3	-0.95
Sdcbp	-0.63
Selenop	-1.52
Selp	-1.57
Sema3c	-0.88
Sema3d	-4.29

Sema3f	-0.55
Serpina1a	-0.57
Serpina1b	-0.53
Serpina1d	-0.58
Serpina3k	-0.47
Serpina3n	-0.46
Serpinb8	-0.46
Serpinc1	-0.42
Serpind1	-0.75
Serpine1	-1.08
Serpine2	-0.97
Serpinf2	-0.98
Serpini1	-0.79
Sfrp1	-0.42
Sfrp2	-0.53
Smpdl3b	-0.45
Sod3	-0.84
Sorl1	-0.41
Spon2	-0.58
Srpx2	-0.64
Sulf1	-0.54
Sulf2	-0.89
Tgfb1	-0.61
Tgfbi	-0.59
Thbd	-0.64
Thbs3	-0.71

Timp2	-0.41
Timp3	-0.67
Tnxb	-1.06
Trf	-0.44
Ttr	-0.47
Vcan	-0.46
Vtn	-1.07
Vwf	-0.48

Supplementary Table S23. The table shows proteins and their log_2 fold change that were enriched in the extracellular matrix.

**Protein**	**Log2 Fold Change**
Abi3bp	-0.88
Acan	-0.60
Adamts1	-1.58
Adamtsl4	-0.48
Adamtsl5	-0.46
Agrn	-0.72
Ahsg	-0.49
Ang	-0.44
Bgn	-0.41
Bmp1	-1.47
Ccdc80	-0.70
Ccn1	-0.69
Ccn2	-0.68
Chad	-0.47

Col15a1	-0.82
Col18a1	-0.66
Col4a6	-0.96
Col6a1	-0.78
Col6a2	-0.73
Col6a3	-0.70
Col6a6	-0.68
Ecm1	-0.72
Ecm2	-0.52
Efemp2	-0.43
Efna5	-0.42
F13a1	-0.44
F2	-0.65
Gp1ba	-1.85
Gpc1	-0.43
Gpc4	-0.44
Gpc6	-0.64
Hspg2	-0.55
Htra1	-0.94
Igfbp6	-0.60
Igfbp7	-0.65
Itih1	-0.60
Itih4	-0.69
Lama2	-0.45
Lama4	-0.40
Lamb2	-0.45

Lamc1	-0.36
Lgals3	-0.49
Loxl1	-0.59
Loxl2	-0.79
Lrrc17	-1.24
Ltbp3	-0.45
Ltbp4	-0.56
Mamdc2	-1.44
Matn2	-0.79
Mbl2	-0.64
Mfap5	-0.50
Mfge8	-0.75
Mgp	-0.80
Mmp17	-0.81
Mmp2	-0.40
Mmrn1	-1.31
Npnt	-0.54
Ntn1	-1.24
Pcolce2	-0.95
Pf4	-1.23
Plg	-0.73
Plxdc2	-0.53
Postn	-0.98
Prelp	-0.40
Sema3c	-0.88
Serpina3k	-0.47

Serpinc1	-0.42
Serpine1	-1.08
Serpine2	-0.97
Serpinf2	-0.98
Sfrp1	-0.42
Smoc1	-0.95
Sod3	-0.84
Spon1	-0.47
Spon2	-0.58
Srpx2	-0.64
Tgfb1	-0.61
Tgfbi	-0.59
Thsd4	-0.66
Timp2	-0.41
Timp3	-0.67
Tnxb	-1.06
Trf	-0.44
Vcan	-0.46
Vtn	-1.07
Vwf	-0.48

Supplementary Table S24. The table shows proteins and their $\log_2$ fold change that were enriched in the extracellular region part.

**Protein**	**Log2 Fold Change**
Abi3bp	-0.88
Acan	-0.60

Ace	-0.83
Adamts1	-1.58
Adamtsl4	-0.48
Adamtsl5	-0.46
Aebp1	-0.79
Agrn	-0.72
Ahsg	-0.49
Ang	-0.44
Angpt2	-1.65
Angptl2	-0.49
Apoa1	-0.54
Apoa4	-0.65
App	-1.03
Atrn	-0.51
Azgp1	-0.42
Bche	-0.65
Bgn	-0.41
Bmp1	-1.47
Bmp3	-1.74
C2	-0.56
C3	-0.48
C8a	-0.52
C8b	-0.53
C8g	-0.65
C9	-0.46
Ccdc80	-0.70

Ccl9	-0.44
Ccn1	-0.69
Ccn2	-0.68
Cd109	-1.12
Cd9	-1.24
Ces2e	-0.94
Cfb	-0.72
Cfh	-0.73
Cfi	-0.64
Chad	-0.47
Clec11a	-0.44
Clstn1	-0.70
Clu	-0.73
Col15a1	-0.82
Col18a1	-0.66
Col4a6	-0.96
Col6a1	-0.78
Col6a2	-0.73
Col6a3	-0.70
Col6a6	-0.68
Cpn1	-0.76
Crp	-1.00
Cyp4a12b	-0.67
Dkk3	-0.51
Ecm1	-0.72
Ecm2	-0.52

Efemp2	-0.43
Efna5	-0.42
F10	-0.64
F13a1	-0.44
F2	-0.65
F5	-2.89
Fetub	-0.48
Fstl1	-0.46
Gas6	-0.61
Gc	-0.52
Gp1ba	-1.85
Gpc1	-0.43
Gpc4	-0.44
Gpc6	-0.64
Gpx3	-0.57
Hspg2	-0.55
Htra1	-0.94
Igfbp5	-0.49
Igfbp6	-0.60
Igfbp7	-0.65
Inhca	-0.50
Itgbl1	-0.64
Itih1	-0.60
Itih4	-0.69
Lama2	-0.45
Lama4	-0.40

Lamb2	-0.45
Lamc1	-0.36
Lgals3	-0.49
Loxl1	-0.59
Loxl2	-0.79
Loxl3	-0.69
Lrrc17	-1.24
Ltbp3	-0.45
Ltbp4	-0.56
Mamdc2	-1.44
Masp1	-1.47
Masp2	-0.82
Matn2	-0.79
Mbl1	-0.96
Mbl2	-0.64
Mfap5	-0.50
Mfge8	-0.75
Mgp	-0.80
Mmp17	-0.81
Mmp2	-0.40
Mmrn1	-1.31
Mst1	-0.48
Mug1	-0.71
Npnt	-0.54
Ntn1	-1.24
Pam	-0.52

Pamr1	-0.56
Pcif1	-0.45
Pcolce2	-0.95
Pf4	-1.23
Pkd1	-0.40
Plg	-0.73
Pltp	-0.43
Plxdc2	-0.53
Postn	-0.98
Ppp1r13l	-0.55
Prelp	-0.40
Pros1	-1.00
Prss23	-0.46
Qsox1	-0.60
Rbp4	-0.43
Rnase4	-0.65
Scube3	-0.95
Sdcbp	-0.63
Selenop	-1.52
Selp	-1.57
Sema3c	-0.88
Sema3d	-4.29
Sema3f	-0.55
Serpina1a	-0.57
Serpina1b	-0.53
Serpina1d	-0.58

Serpina3k	-0.47
Serpina3n	-0.46
Serpinb8	-0.46
Serpinc1	-0.42
Serpind1	-0.75
Serpine1	-1.08
Serpine2	-0.97
Serpinf2	-0.98
Serpini1	-0.79
Sfrp1	-0.42
Sfrp2	-0.53
Smoc1	-0.95
Smpdl3b	-0.45
Sod3	-0.84
Sorl1	-0.41
Spon1	-0.47
Spon2	-0.58
Srpx2	-0.64
Sulf1	-0.54
Sulf2	-0.89
Tgfb1	-0.61
Tgfbi	-0.59
Thbd	-0.64
Thbs3	-0.71
Thsd4	-0.66
Timp2	-0.41

Timp3	-0.67
Tnxb	-1.06
Trf	-0.44
Ttr	-0.47
Vcan	-0.46
Vtn	-1.07
Vwf	-0.48

Supplementary Table S25. The table shows proteins and their log_2 fold change that were enriched in the collagen-containing extracellular matrix.

**Protein**	**Log2 Fold Change**
Abi3bp	-0.88
Acan	-0.60
Adamts1	-1.58
Adamtsl4	-0.48
Adamtsl5	-0.46
Agrn	-0.72
Ahsg	-0.49
Ang	-0.44
Bgn	-0.41
Bmp1	-1.47
Ccdc80	-0.70
Col15a1	-0.82
Col18a1	-0.66
Col4a6	-0.96
Col6a1	-0.78

Col6a2	-0.73
Col6a3	-0.70
Col6a6	-0.68
Ecm1	-0.72
Ecm2	-0.52
Efemp2	-0.43
Efna5	-0.42
F13a1	-0.44
F2	-0.65
Gpc1	-0.43
Gpc4	-0.44
Gpc6	-0.64
Hspg2	-0.55
Htra1	-0.94
Igfbp6	-0.60
Igfbp7	-0.65
Itih1	-0.60
Itih4	-0.69
Lama2	-0.45
Lama4	-0.40
Lamb2	-0.45
Lamc1	-0.36
Lgals3	-0.49
Loxl1	-0.59
Loxl2	-0.79
Ltbp4	-0.56

Mamdc2	-1.44
Matn2	-0.79
Mfap5	-0.50
Mfge8	-0.75
Mgp	-0.80
Mmrn1	-1.31
Npnt	-0.54
Ntn1	-1.24
Pcolce2	-0.95
Pf4	-1.23
Plg	-0.73
Plxdc2	-0.53
Postn	-0.98
Prelp	-0.40
Sema3c	-0.88
Serpina3k	-0.47
Serpinc1	-0.42
Serpine1	-1.08
Serpine2	-0.97
Serpinf2	-0.98
Sfrp1	-0.42
Smoc1	-0.95
Sod3	-0.84
Spon1	-0.47
Srpx2	-0.64
Tgfb1	-0.61

Tgfbi	-0.59
Timp2	-0.41
Timp3	-0.67
Tnxb	-1.06
Trf	-0.44
Vcan	-0.46
Vtn	-1.07
Vwf	-0.48

Supplementary Table S26. The table shows proteins and their log_2 fold change that were enriched in the basement membrane.

**Protein**	**Log2 Fold Change**
Acan	-0.60
Adamts1	-1.58
Agrn	-0.72
Ang	-0.44
Ccdc80	-0.70
Col15a1	-0.82
Col18a1	-0.66
Col4a6	-0.96
Efemp1	-0.32
Efna5	-0.42
Hspg2	-0.55
Lama2	-0.45
Lama4	-0.40
Lamb2	-0.45

Lamc1	-0.36
Loxl1	-0.59
Loxl2	-0.79
Matn2	-0.79
Npnt	-0.54
Ntn1	-1.24
Papln	-0.35
Serpinf1	-0.36
Smoc1	-0.95
Sparc	-0.32
Tgfb2	-0.31
Tgfbi	-0.59
Timp2	-0.41
Timp3	-0.67
Trf	-0.44
Vtn	-1.07
Vwa1	-0.38

Supplementary Table S27. The table shows proteins and their log_2 fold change that were enriched in cell projection.

**Protein**	**Log2 Fold Change**
Aak1	-0.22
Ablim1	-0.34
Acvrl1	-0.35
Adam10	-0.34
Asap1	-0.33

Aspn	-0.36
Atp2b4	-0.35
Bcar1	-0.39
Bmpr2	-0.33
Cd44	-0.40
Cdh13	-0.33
Cep170	-0.31
Cnn3	-0.34
Cpe	-0.33
Creb1	-0.37
Ctsl	-0.31
Diaph1	-0.35
Dmtn	-0.34
Dpysl5	-0.32
Dtna	-0.31
Dysf	-0.31
Fus	-0.36
Gabra3	-0.37
Gja5	-0.35
Igsf8	-0.33
Itga3	-0.39
Itga8	-0.35
Itgav	-0.38
Ldlrap1	-0.34
Lrp1	-0.35
Mfsd10	-0.37

Nptn	-0.35
Nradd	-0.32
Osbpl3	-0.35
Pdlim4	-0.31
Phactr4	-0.33
Ptgs1	-0.39
Ptprs	-0.31
Pxn	-0.38
Rasgrp2	-0.34
Rilp	-0.33
Serpinf1	-0.36
Sgce	-0.35
Sh2b1	-0.36
Slc9a6	-0.31
Snap29	-0.35
Src	-0.32
Tgfb2	-0.31
Wipf1	-0.35

Supplementary Table S28. The table shows proteins and their log_2 fold change that were enriched in plasma membrane-bound cell projection.

Protein	Log2 Fold Change
Acvrl1	-0.35
Adam10	-0.34
Bmpr2	-0.33
Cd44	-0.40

Cdh13	-0.33
Cpe	-0.33
Creb1	-0.37
Bcar1	-0.39
Ctsl	-0.31
Asap1	-0.33
Diaph1	-0.35
Dtna	-0.31
Dmtn	-0.34
Gabra3	-0.37
Itga3	-0.39
Itgav	-0.38
Lrp1	-0.35
Ptgs1	-0.39
Ptprs	-0.31
Pxn	-0.38
Rasgrp2	-0.34
Serpinf1	-0.36
Nptn	-0.35
Sgce	-0.35
Sh2b1	-0.36
Src	-0.32
Tgfb2	-0.31
Dysf	-0.31
Pdlim4	-0.31
Dpysl5	-0.32

Nradd	-0.32
Snap29	-0.35
Mfsd10	-0.37
Cnn3	-0.34
Ldlrap1	-0.34
Phactr4	-0.33
Igsf8	-0.33
Ablim1	-0.34
Fus	-0.36
Slc9a6	-0.31
Itga8	-0.35
Aak1	-0.22
Rilp	-0.33
Atp2b4	-0.35
Cep170	-0.31

Supplementary Table S29. The table shows proteins and their log_2 fold change that were enriched in nucleosomes.

**Protein**	**Log2 Fold Change**
H4c1	-0.22
H4c11	-0.22
H4c12	-0.22
H4c14	-0.22
H4c16	-0.22
H4c17	-0.22
H4c18	-0.22

H4c2	-0.22
H4c3	-0.22
H4c4	-0.22
H4c6	-0.22
H4c8	-0.22
H4c9	-0.22

Supplementary Table S30. The table shows proteins and their log_2 fold change that were enriched in nuclear nucleosomes.

**Protein**	**Log2 Fold Change**
H4c1	-0.22
H4c11	-0.22
H4c12	-0.22
H4c14	-0.22
H4c16	-0.22
H4c17	-0.22
H4c18	-0.22
H4c2	-0.22
H4c3	-0.22
H4c4	-0.22
H4c6	-0.22
H4c8	-0.22
H4c9	-0.22

Supplementary Table S31. The table shows proteins and their log_2 fold change that were enriched in the extracellular matrix structural constituent conferring tensile strength.

Protein	Log2 Fold Change
Col15a1	-0.824
Col18a1	-0.66
Col4a6	-0.96
Col6a1	-0.778
Col6a2	-0.728
Col6a3	-0.7
Col6a6	-0.684

Supplementary Table S32. The table shows proteins and their log_2 fold change that were enriched in serine-type endopeptidase activity.

Protein	Log2 Fold Change
C2	-0.564
Cfb	-0.722
Cfi	-0.644
F10	-0.642
F2	-0.652
Htra1	-0.94
Htra3	-0.904
Htra4	-1.382
Masp1	-1.47
Masp2	-0.824
Mmp2	-0.404
Mst1	-0.484
Pamr1	-0.558
Plg	-0.732

Prss23	-0.462

Supplementary Table S33. The table shows proteins and their log_2 fold change that were enriched in enzyme inhibitor activity.

Protein	Log2 Fold Change
Agrn	-0.724
Ahsg	-0.492
Apoa1	-0.542
App	-1.034
C3	-0.476
Cabin1	-1.066
Cd109	-1.118
F13b	-1.272
Fetub	-0.484
Gas6	-0.606
Inhca	-0.496
Itih1	-0.598
Itih4	-0.688
Itprip	-0.442
Mgat5	-0.434
Mug1	-0.712
Serpina1a	-0.57
Serpina1b	-0.528
Serpina1d	-0.578
Serpina3k	-0.474
Serpina3n	-0.464

Serpinb8	-0.462
Serpinc1	-0.418
Serpind1	-0.752
Serpine1	-1.076
Serpine2	-0.966
Serpinf2	-0.978
Serpini1	-0.788
Timp2	-0.414
Timp3	-0.666

Supplementary Table S34. The table shows proteins and their log_2 fold change that were enriched in endopeptidase inhibitor activity.

Protein	Log2 Fold Change
Ahsg	-0.83
App	-1.58
C3	-0.79
Cd109	-0.60
Fetub	-1.65
Gas6	-0.72
Itih1	-0.49
Itih4	-0.44
Mug1	-0.54
Serpina1a	-0.65
Serpina1b	-1.03
Serpina1d	-0.42
Serpina3k	-0.51

Serpina3n	-0.42
Serpinb8	-0.65
Serpinc1	-0.41
Serpind1	-1.47
Serpine1	-0.56
Serpine2	-0.48
Serpinf2	-0.46
Serpini1	-1.24
Timp2	-0.73
Timp3	-0.64

Supplementary Table S35. The table shows proteins and their log_2 fold change that were enriched in the extracellular matrix structural constituent.

**Protein**	**Log2 Fold Change**
Abi3bp	-0.876
Agrn	-0.724
Bgn	-0.41
Col15a1	-0.824
Col18a1	-0.66
Col4a6	-0.96
Col6a1	-0.778
Col6a2	-0.728
Col6a3	-0.7
Col6a6	-0.684
Ecm1	-0.72
Efemp2	-0.43

Hspg2	-0.548
Igfbp6	-0.596
Igfbp7	-0.646
Lama2	-0.452
Lama4	-0.402
Lamb2	-0.446
Lamc1	-0.358
Ltbp4	-0.562
Matn2	-0.786
Mfap5	-0.496
Mfge8	-0.75
Mmrn1	-1.308
Npnt	-0.542
Ntn1	-1.242
Pcolce2	-0.946
Postn	-0.978
Prelp	-0.4
Spon1	-0.466
Srpx2	-0.636
Tgfbi	-0.594
Tnxb	-1.056
Vcan	-0.462
Vtn	-1.074
Vwf	-0.482

Supplementary Table S36. The table shows proteins and their $\log_2$ fold change that were enriched in glycosaminoglycan binding.

Protein	Log2 Fold Change
Abi3bp	-0.876
Acan	-0.602
Adamts1	-1.58
Adamtsl5	-0.456
Agrn	-0.724
Ang	-0.438
Aplp2	-0.606
App	-1.034
Bgn	-0.41
Ccdc80	-0.704
Ccn1	-0.69
Ccn2	-0.676
Cfh	-0.732
Ecm2	-0.518
F2	-0.652
Fstl1	-0.462
Mamdc2	-1.44
Pcolce2	-0.946
Pf4	-1.23
Postn	-0.978
Prelp	-0.4
Selp	-1.574
Serpinc1	-0.418
Serpind1	-0.752
Serpine2	-0.966

Sfrp1	-0.424
Sulf1	-0.542
Sulf2	-0.886
Thbs3	-0.714
Tnxb	-1.056
Vcan	-0.462
Vtn	-1.074

Supplementary Table S37. The table shows proteins and their log_2 fold change that were enriched in heparin binding.

**Protein**	**Log2 Fold Change**
Abi3bp	-0.876
Adamts1	-1.58
Adamtsl5	-0.456
Ang	-0.438
Aplp2	-0.606
App	-1.034
Ccdc80	-0.704
Ccn1	-0.69
Ccn2	-0.676
Cfh	-0.732
Ecm2	-0.518
F2	-0.652
Fstl1	-0.462
Pcolce2	-0.946
Pf4	-1.23

Postn	-0.978
Prelp	-0.4
Selp	-1.574
Serpinc1	-0.418
Serpind1	-0.752
Serpine2	-0.966
Sfrp1	-0.424
Thbs3	-0.714
Tnxb	-1.056
Vtn	-1.074

Supplementary Table S38. The table shows proteins and their $\log_2$ fold change that were enriched in serine-type peptidase activity.

**Protein**	**Log2 Fold Change**
Ace	-0.834
C2	-0.564
Cfb	-0.722
Cfi	-0.644
F10	-0.642
F2	-0.652
Htra1	-0.94
Htra3	-0.904
Htra4	-1.382
Masp1	-1.47
Masp2	-0.824
Mmp2	-0.404

Mst1	-0.484
Pamr1	-0.558
Plg	-0.732
Prss23	-0.462

Supplementary Table S39. The table shows proteins and their log_2 fold change that were enriched in serine hydrolase activity.

Protein	Log2 Fold Change
Ace	-0.834
C2	-0.564
Cfb	-0.722
Cfi	-0.644
F10	-0.642
F2	-0.652
Htra1	-0.94
Htra3	-0.904
Htra4	-1.382
Masp1	-1.47
Masp2	-0.824
Mmp2	-0.404
Mst1	-0.484
Pamr1	-0.558
Plg	-0.732
Prss23	-0.462

Supplementary Table S40. The table shows proteins and their log_2 fold change that were enriched in peptidase inhibitor activity.

Protein	Log2 Fold Change
Ahsg	-0.492
App	-1.034
C3	-0.476
Cd109	-1.118
Fetub	-0.484
Gas6	-0.606
Itih1	-0.598
Itih4	-0.688
Mug1	-0.712
Serpina1a	-0.57
Serpina1b	-0.528
Serpina1d	-0.578
Serpina3k	-0.474
Serpina3n	-0.464
Serpinb8	-0.462
Serpinc1	-0.418
Serpind1	-0.752
Serpine1	-1.076
Serpine2	-0.966
Serpinf2	-0.978
Serpini1	-0.788
Timp2	-0.414
Timp3	-0.666

Supplementary Table S41. The table shows proteins and their log_2 fold change that were enriched in blood coagulation.

Protein	Log2 Fold Change
Cd9	-1.24
F10	-0.64
F13b	-1.27
F2	-0.65
F5	-2.89
Gp1ba	-1.85
Gp1bb	-1.88
Gp5	-1.92
Serpind1	-0.75
Serpine1	-1.08
Plg	-0.73
Serpinf2	-0.98
Pros1	-1.00
Selp	-1.57
Serpine2	-0.97
Thbd	-0.64
Pf4	-1.23
Mmrn1	-1.31
Treml1	-1.80
Mpig6b	-3.05

Supplementary Table S42. The table shows proteins and their log_2 fold change that were enriched in hemostasis.

Protein	Log2 Fold Change
Cd9	-1.24

F10	-0.64
F13b	-1.27
F2	-0.65
F5	-2.89
Gp1ba	-1.85
Gp1bb	-1.88
Gp5	-1.92
Serpind1	-0.75
Serpine1	-1.08
Plg	-0.73
Serpinf2	-0.98
Pros1	-1.00
Selp	-1.57
Serpine2	-0.97
Thbd	-0.64
Pf4	-1.23
Mmrn1	-1.31
Treml1	-1.80
Mpig6b	-3.05

Supplementary Table S43. The table shows proteins and their log_2 fold change that were enriched in the regulation of blood coagulation.

**Protein**	**Log2 Fold Change**
Cd9	-1.24
F2	-0.65
Gp1ba	-1.85

Gp5	-1.92
Serpine1	-1.08
Plg	-0.73
Serpinf2	-0.98
Pros1	-1.00
Selp	-1.57
Serpine2	-0.97
Thbd	-0.64

Supplementary Table S44. The table shows proteins and their log_2 fold change that were enriched in the negative regulation of blood coagulation.

**Protein**	**Log2 Fold Change**
Cd9	-1.24
F2	-0.65
Gp1ba	-1.85
Gp5	-1.92
Serpine1	-1.08
Plg	-0.73
Serpinf2	-0.98
Pros1	-1.00
Serpine2	-0.97
Thbd	-0.64

Supplementary Table S45. The table shows proteins and their log_2 fold change that were enriched in coagulation.

**Protein**	**Log2 Fold Change**

Cd9	-1.24
F10	-0.64
F13b	-1.27
F2	-0.65
F5	-2.89
Gp1ba	-1.85
Gp1bb	-1.88
Gp5	-1.92
Serpind1	-0.75
Serpine1	-1.08
Plg	-0.73
Serpinf2	-0.98
Pros1	-1.00
Selp	-1.57
Serpine2	-0.97
Thbd	-0.64
Pf4	-1.23
Mmrn1	-1.31
Treml1	-1.80
Mpig6b	-3.05

Supplementary Table S46. The table shows proteins and their $\log_2$ fold change that were enriched in the regulation of coagulation.

**Protein**	**Log2 Fold Change**
Cd9	-1.24
F2	-0.65

Gp1ba	-1.85
Gp5	-1.92
Serpine1	-1.08
Plg	-0.73
Serpinf2	-0.98
Pros1	-1.00
Selp	-1.57
Serpine2	-0.97
Thbd	-0.64

Supplementary Table S47. The table shows proteins and their log_2 fold change that were enriched in the negative regulation of coagulation.

**Protein**	**Log2 Fold Change**
Cd9	-1.24
F2	-0.65
Gp1ba	-1.85
Gp5	-1.92
Serpine1	-1.08
Plg	-0.73
Serpinf2	-0.98
Pros1	-1.00
Serpine2	-0.97
Thbd	-0.64

Supplementary Table S48. The table shows proteins and their log_2 fold change that were enriched in the regulation of body fluid levels.

Protein	Log2 Fold Change
Cd9	-1.24
F10	-0.64
F13b	-1.27
F2	-0.65
F5	-2.89
Gp1ba	-1.85
Gp1bb	-1.88
Gp5	-1.92
Serpind1	-0.75
Serpine1	-1.08
Plg	-0.73
Serpinf2	-0.98
Pros1	-1.00
Selp	-1.57
Serpine2	-0.97
Thbd	-0.64
Pf4	-1.23
Mmrn1	-1.31
Treml1	-1.80
Mpig6b	-3.05

Supplementary Table S49. The table shows proteins and their log_2 fold change that were enriched in the negative regulation of wound healing.

Protein	Log2 Fold Change
Cd9	-1.24

F2	-0.65
Gp1ba	-1.85
Gp5	-1.92
Serpine1	-1.08
Plg	-0.73
Serpinf2	-0.98
Pros1	-1.00
Serpine2	-0.97
Thbd	-0.64
Cd109	-1.12

Supplementary Table S50. The table shows proteins and their log_2 fold change that were enriched in the regulation of hemostasis.

**Protein**	**Log2 Fold Change**
Cd9	-1.24
F2	-0.65
Gp1ba	-1.85
Gp5	-1.92
Serpine1	-1.08
Plg	-0.73
Serpinf2	-0.98
Pros1	-1.00
Selp	-1.57
Serpine2	-0.97
Thbd	-0.64

Supplementary Table S51. The table shows proteins and their log_2 fold change that were enriched in the negative regulation of hemostasis.

Protein	Log2 Fold Change
Cd9	-1.24
F2	-0.65
Gp1ba	-1.85
Gp5	-1.92
Serpine1	-1.08
Plg	-0.73
Serpinf2	-0.98
Pros1	-1.00
Serpine2	-0.97
Thbd	-0.64

Supplementary Table S52. The table shows proteins and their log_2 fold change that were enriched in the negative regulation of response to wounding.

Protein	Log2 Fold Change
Cd9	-1.24
F2	-0.65
Gp1ba	-1.85
Gp5	-1.92
Serpine1	-1.08
Plg	-0.73
Serpinf2	-0.98
Pros1	-1.00
Serpine2	-0.97

Thbd	-0.64
Cd109	-1.12

Supplementary Table S53. The table shows proteins and their log_2 fold change that were enriched in organellar ribosomes.

**Protein**	**Log2 Fold Change**
Mrpl13	0.36
Mrpl44	0.37
Mrps30	0.37
Mrpl4	0.37
Mrpl58	0.38
Mrps31	0.43
Mrps21	0.43
Mrpl46	0.45
Mrpl27	0.48
Mrpl34	0.49
Mrpl38	0.52
Mrpl19	0.52
Mrpl41	0.53
Mrpl14	0.55
Mrpl22	0.56
Ndufa7	0.57
Mrpl40	0.57
Mrpl17	0.78
Chchd1	0.84
Mrpl12	1.07

Supplementary Table S54. The table shows proteins and their $\log_2$ fold change that were enriched in an organellar large ribosomal subunit.

Protein	Log2 Fold Change
Mrpl13	0.36
Mrpl44	0.37
Mrps30	0.37
Mrpl4	0.37
Mrpl58	0.38
Mrpl46	0.45
Mrpl27	0.48
Mrpl34	0.49
Mrpl38	0.52
Mrpl19	0.52
Mrpl41	0.53
Mrpl14	0.55
Mrpl22	0.56
Mrpl40	0.57
Mrpl17	0.78
Mrpl12	1.07

Supplementary Table S55. The table shows proteins and their $\log_2$ fold change that were enriched in the mitochondrial respiratory chain.

Protein	Log2 Fold Change
Cyc1	0.41
Ndufa12	0.42
Ndufa10	0.45

Ndufs7	0.46
Ndufa7	0.57
Cox5b	0.57
Ndufb7	0.58
Cox4i1	0.64
Ndufb8	0.66
Ndufv2	0.69
Ndufv3	0.78
Ndufb5	0.80
Ndufb4	0.80
Uqcrb	0.89

Supplementary Table S56. The table shows proteins and their log_2 fold change that were enriched in the mitochondrial matrix.

**Protein**	**Log2 Fold Change**
Mrpl13	0.36
Ivd	0.36
Mrpl44	0.37
Mrps30	0.37
Mrpl4	0.37
Cbr4	0.37
Mrpl58	0.38
Pam16	0.38
Tsfm	0.40
Hsd17b8	0.42
Hsd17b10	0.42

Mrps31	0.43
Mrps21	0.43
Grpel1	0.44
Mrpl46	0.45
Rida	0.47
Mrpl27	0.48
Mrpl34	0.49
Mdh2	0.50
Mrpl38	0.52
Mrpl19	0.52
Mrpl41	0.53
Dtymk	0.55
Mrpl14	0.55
Polg2	0.55
Slc25a5	0.56
Mrpl22	0.56
Ndufa7	0.57
Clpp	0.57
Mrpl40	0.57
Gstk1	0.59
Sod2	0.64
Ak3	0.66
Mrpl17	0.78
Chchd1	0.84
Hmgcs2	0.88
Acss1	0.96

Mrpl12	1.07
Ak4	1.34

Supplementary Table S57. The table shows proteins and their log_2 fold change that were enriched in mitochondrial ribosomes.

**Protein**	**Log2 Fold Change**
Mrpl13	0.36
Mrpl44	0.37
Mrps30	0.37
Mrpl4	0.37
Mrpl58	0.38
Mrps31	0.43
Mrps21	0.43
Mrpl46	0.45
Mrpl27	0.48
Mrpl34	0.49
Mrpl38	0.52
Mrpl19	0.52
Mrpl41	0.53
Mrpl14	0.55
Mrpl22	0.56
Ndufa7	0.57
Mrpl40	0.57
Mrpl17	0.78
Chchd1	0.84
Mrpl12	1.07

Supplementary Table S58. The table shows proteins and their log_2 fold change that were enriched in mitochondrial large ribosomal subunits.

Protein	Log2 Fold Change
Mrpl13	0.36
Mrpl44	0.37
Mrps30	0.37
Mrpl4	0.37
Mrpl58	0.38
Mrpl46	0.45
Mrpl27	0.48
Mrpl34	0.49
Mrpl38	0.52
Mrpl19	0.52
Mrpl41	0.53
Mrpl14	0.55
Mrpl22	0.56
Mrpl40	0.57
Mrpl17	0.78
Mrpl12	1.07

Supplementary Table S59. The table shows proteins and their log_2 fold change that were enriched in mitochondrial parts.

Protein	Log2 Fold Change
Mrpl13	0.36
Ivd	0.36
Mrpl44	0.37

Mrps30	0.37
Chchd4	0.37
Mrpl4	0.37
Cbr4	0.37
Mrpl58	0.38
Slc25a3	0.38
Pam16	0.38
Tsfm	0.40
Atp5po	0.41
Cyc1	0.41
Ndufaf3	0.42
Ndufa12	0.42
Hsd17b8	0.42
Hsd17b10	0.42
Mrps31	0.43
Mrps21	0.43
Bax	0.43
Grpel1	0.44
Mrpl46	0.45
Slc25a40	0.45
Ndufa10	0.45
Endog	0.45
Ndufs7	0.46
Cytb	0.46
Atp5mf	0.46
Rida	0.47

Nipsnap2	0.47
Mrpl27	0.48
Mrpl34	0.49
Apoo	0.49
Mdh2	0.50
Mrpl38	0.52
Mrpl19	0.52
Mrpl41	0.53
Slc25a11	0.54
Dmpk	0.54
Slc25a4	0.54
Dtymk	0.55
Mrpl14	0.55
Polg2	0.55
Slc25a5	0.56
Mrpl22	0.56
Ndufa7	0.57
Clpp	0.57
Cox5b	0.57
Mrpl40	0.57
Ndufb7	0.58
Gstk1	0.59
Atp5me	0.63
Sod2	0.64
Cox4i1	0.64
Atp5pb	0.65

Ndufb8	0.66
Ak3	0.66
Ndufv2	0.69
Mcur1	0.73
Ndufv3	0.78
Mrpl17	0.78
Ndufb5	0.80
Ndufb4	0.80
Chchd1	0.84
Ndufaf5	0.87
Hmgcs2	0.88
Uqcrb	0.89
Fis1	0.91
Acss1	0.96
Mrpl12	1.07
Mrs2	1.08
Atp5pf	1.10
Ak4	1.34
Arl2bp	2.40

Supplementary Table S60. The table shows proteins and their log_2 fold change that were enriched in mitochondrial membrane parts.

**Protein**	**Log2 Fold Change**
Slc25a3	0.38
Pam16	0.38
Atp5po	0.41

Cyc1	0.41
Ndufa12	0.42
Grpel1	0.44
Ndufa10	0.45
Ndufs7	0.46
Cytb	0.46
Atp5mf	0.46
Apoo	0.49
Dmpk	0.54
Ndufa7	0.57
Cox5b	0.57
Ndufb7	0.58
Atp5me	0.63
Cox4i1	0.64
Atp5pb	0.65
Ndufb8	0.66
Ndufv2	0.69
Mcur1	0.73
Ndufv3	0.78
Ndufb5	0.80
Ndufb4	0.80
Ndufaf5	0.87
Uqcrb	0.89
Fis1	0.91
Atp5pf	1.10

Supplementary Table S61. The table shows proteins and their log_2 fold change that were enriched in the respiratory chain.

Protein	Log2 Fold Change
Cyc1	0.41
Ndufa12	0.42
Ndufa10	0.45
Ndufs7	0.46
Cytb	0.46
Ndufa7	0.57
Cox5b	0.57
Ndufb7	0.58
Cox4i1	0.64
Ndufb8	0.66
Ndufv2	0.69
Ndufv3	0.78
Ndufb5	0.80
Ndufb4	0.80
Uqcrb	0.89

Supplementary Table S62. The table shows proteins and their log_2 fold change that were enriched in inner mitochondrial membrane protein complexes.

Protein	Log2 Fold Change
Pam16	0.38
Atp5po	0.41
Cyc1	0.41
Ndufa12	0.42

Grpel1	0.44
Ndufa10	0.45
Ndufs7	0.46
Atp5mf	0.46
Apoo	0.49
Ndufa7	0.57
Cox5b	0.57
Ndufb7	0.58
Atp5me	0.63
Cox4i1	0.64
Atp5pb	0.65
Ndufb8	0.66
Ndufv2	0.69
Ndufv3	0.78
Ndufb5	0.80
Ndufb4	0.80
Uqcrb	0.89
Atp5pf	1.10

Supplementary Table S63. The table shows proteins and their log_2 fold change that were enriched in respiratory chain complexes.

Protein	Log2 Fold Change
Cyc1	0.41
Ndufa12	0.42
Ndufa10	0.45
Ndufs7	0.46

Cytb	0.46
Ndufa7	0.57
Cox5b	0.57
Ndufb7	0.58
Cox4i1	0.64
Ndufb8	0.66
Ndufv2	0.69
Ndufv3	0.78
Ndufb5	0.80
Ndufb4	0.80
Uqcrb	0.89

Supplementary Table S64. The table shows proteins and their log_2 fold change that were enriched in oxidoreductase complexes.

**Protein**	**Log2 Fold Change**
Cbr4	0.37
Cyc1	0.41
Ndufa12	0.42
Ndufa10	0.45
Ndufs7	0.46
Cytb	0.46
Ndufa7	0.57
Ndufb7	0.58
Ndufb8	0.66
Ndufv2	0.69
Ndufv3	0.78

Ndufb5	0.80
Ndufb4	0.80
Uqcrb	0.89

Supplementary Table S65. Expression of proteins downregulated in both Myh11$^{\Delta K/\Delta K}$ aortas and zyxin knockout VSMCs after stretch stimulation.

Protein	Log2 Fold Change
Aoc3	-0.292
C2	-0.564
Cfi	-0.644
Epn1	-0.16
Fkbp7	-0.204
Frg1	-0.208
Gbp2	-0.144
Itga5	-0.558
Slc3a2	-0.25
Mfge8	-0.75
Foxk1	-0.16
Numbl	-0.142
Pdgfrb	-0.124
Rab23	-0.156
Rasa3	-0.384
Nptn	-0.346
Sin3b	-0.152
Sdc4	-0.42
Angptl2	-0.494

Adgre5	-0.454
Igfbp7	-0.646
Tor3a	-0.194
Snrnp27	-0.282
Dnajc10	-0.338
Tbc1d20	-0.124
Wls	-0.192
Pdgfrl	-0.938
Ctc1	-0.196
Antxr1	-0.266
Rab43	-1.074
Thap12	-0.294
Wasl	-0.474
Dnm1l	-0.172
Uvrag	-0.256
Avl9	-0.312
Stk17b	-0.674
Rab28	-0.248
Pi4kb	-0.2
Slc8a2	-0.16
Cd99l2	-0.234
Zfp280c	-0.268
Ttc28	-0.248
Cfap36	-0.244
Fcho2	-0.408
Lims2	-0.164

Mavs	-0.36
Prr14	-0.198
Fez1	-1.508
Slc9a6	-0.314
Vwa1	-0.376
Mical2	-0.15
Prr14	-0.198
Fez1	-1.508
Slc9a6	-0.314
Vwa1	-0.376
Mical2	-0.15
